# Plasma 25-Hydroxyvitamin D Concentrations are Associated with Polyunsaturated Fatty Acid Metabolites in Young Children: Results from the Vitamin D Antenatal Asthma Reduction Trial

**DOI:** 10.3390/metabo10040151

**Published:** 2020-04-14

**Authors:** Mengna Huang, Rachel S. Kelly, Priyadarshini Kachroo, Su H. Chu, Kathleen Lee-Sarwar, Bo L. Chawes, Hans Bisgaard, Augusto A. Litonjua, Scott T. Weiss, Jessica Lasky-Su

**Affiliations:** 1 Channing Division of Network Medicine, Department of Medicine, Brigham and Women’s Hospital and Harvard Medical School, Boston, MA 02115, USA; mengna.huang@channing.harvard.edu (M.H.); rachel.kelly@channing.harvard.edu (R.S.K.); priyadarshini.kachroo@channing.harvard.edu (P.K.); su.chu@channing.harvard.edu (S.H.C.); klee-sarwar@bwh.harvard.edu (K.L.-S.); scott.weiss@channing.harvard.edu (S.T.W.); 2 Division of Rheumatology, Immunology and Allergy, Brigham and Women’s Hospital and Harvard Medical School, Boston, MA 02115, USA; 3 Copenhagen Prospective Studies on Asthma in Childhood (COPSAC), Herlev and Gentofte Hospital, University of Copenhagen, 2820 Copenhagen, Denmark; chawes@copsac.com (B.L.C.); bisgaard@copsac.com (H.B.); 4 Division of Pediatric Pulmonary Medicine, Department of Pediatrics, Golisano Children’s Hospital at Strong, University of Rochester Medical Center, Rochester, NY 14642, USA; Augusto_Litonjua@urmc.rochester.edu

**Keywords:** metabolomic epidemiology, vitamin D, 25-hydroxyvitamin D, polyunsaturated fatty acids, n-6 polyunsaturated fatty acids, Vitamin D Antenatal Asthma Reduction Trial (VDAART)

## Abstract

Vitamin D deficiency contributes to a multitude of health conditions, but its biological mechanisms are not adequately understood. Untargeted metabolomics offers the opportunity to comprehensively examine the metabolic profile associated with variations in vitamin D concentrations. The objective of the current analysis was to identify metabolites and metabolic pathways associated with plasma 25-hydroxyvitamin D [25(OH)D] concentrations. The current study included children of pregnant women in the Vitamin D Antenatal Asthma Reduction Trial, who had 25(OH)D and global metabolomics data at age 1 and 3 years. We assessed the cross-sectional associations between individual metabolites and 25(OH)D using linear regression adjusting for confounding factors. Twelve metabolites were significantly associated with plasma 25(OH)D concentrations at both age 1 and 3 after correction for multiple comparisons, including three members of the n-6 polyunsaturated fatty acid (PUFA) metabolism pathway (linoleate, arachidonate, and docosapentaenoate) inversely associated with 25(OH)D. These PUFAs along with four other significant metabolites were replicated in the independent Childhood Asthma Management Program (CAMP) cohort. Both vitamin D and n-6 PUFAs are involved in inflammatory processes, and evidence from cell and animal studies demonstrate a plausible biological mechanism where the active form of 25(OH)D may influence n-6 PUFA metabolism. These relationships warrant further investigation in other populations.

## 1. Introduction

Apart from its classic function in calcium homeostasis and bone health [1,2], vitamin D is an important regulator of the immune system, acting in both innate and adaptive immunity [2,3]. This is partially evidenced by the fact that vitamin D receptors are expressed by many types of immune cells, where circulating 25-hydroxyvitamin D [25(OH)D] can be converted to its active form 1,25-dihydroxyvitamin D [1,25(OH)_2_D] locally [4]. Circulating concentrations of 25(OH)D are generally considered the most reliable biochemical marker of vitamin D status in epidemiologic studies, reflecting both vitamin D obtained through diet and vitamin D synthesized in the skin after exposure to ultraviolet sunlight [1,5]. Despite the importance of maintaining a sufficient vitamin D concentration, deficiency in vitamin D remains widespread across the world [3].

Vitamin D is thought to be involved in many immune and chronic inflammatory diseases: low concentrations of circulating vitamin D have been linked to increased risk of asthma and allergy [3,6], autoimmune diseases [2], cardio-metabolic health [7,8], and cancer [9]. For asthma-related phenotypes, vitamin D supplementation during pregnancy has been shown to be protective against offspring asthma or persistent wheezing by age 3 [10]. A recent randomized controlled trial in preterm black infants suggested that postnatal supplementation may be important in decreasing risk of recurrent wheezing by age 1 year [11]. Vitamin D supplementation may also decrease the rate of asthma exacerbations [12]. Several lines of evidence support a mechanistic role of vitamin D in different endotypes of asthma [13].

Untargeted metabolomics is a high-throughput technology whereby large amounts of small molecules in a biospecimen can be characterized and quantified for their relative abundances [14], which may serve as a useful tool to gain a more comprehensive understanding of the biochemical pathways involved in the multifaceted functions of vitamin D. Previous investigations have examined metabolites associated with vitamin D status predominantly in adults [15,16] and populations with existing health conditions [17,18,19]. There is a need for studies targeting the earliest life course prior to development of diseases, where vitamin D concentrations may influence pathogenesis.

Vitamin D status during infancy and early life are of particular importance as the development of the immune system occurs both prenatally and in the first years of life, and has lifelong impact on an individual’s health [20]. Therefore, within the existing structure of the Vitamin D Antenatal Asthma Reduction Trial (VDAART), which randomized pregnant women to receive either 4400 or 400 IU/day of vitamin D supplementation during pregnancy, untargeted metabolomic data were obtained from stored plasma samples of VDAART children collected at age 1 and age 3. The objective of the current analysis was to identify metabolites and metabolic pathways that are associated with plasma 25(OH)D concentrations. Findings from this investigation may provide insight into the underlying mechanism of health conditions affected by altered vitamin D status.

## 2. Results

### 2.1. Results from Age 1 Analysis

Four hundred and sixty-nine children were measured for metabolomics at age 1, of whom 17 (3.6%) were missing plasma 25(OH)D measurements, and two were further missing body mass index (BMI). As a result, a total of 450 children at age 1 had complete information and were included in the analysis (Table 1). The average plasma 25(OH)D concentration at age 1 was 29.6 ng/mL (standard deviation (SD) = 8.4 ng/mL). One hundred and ninety-six (43.5%) children had plasma 25(OH)D concentrations > 30 ng/mL. Overall white children, non-Hispanic-or-Latino children, and those from the St. Louis site had lower plasma 25(OH)D concentrations. 

From the primary linear model, 19 out of 511 metabolites investigated had significant associations with plasma 25(OH)D concentration after Bonferroni correction (*P*-value threshold = 9.78 × 10^−5^), adjusting for sex, race, ethnicity, study site, age 1, BMI, season of blood collection, and asthma or recurrent wheezing status by age 3. The smallest *P*-value was 1.93 × 10^−8^ for the association between docosadienoate (22:2 n-6) and 25(OH)D, with a negative direction of effect (estimated β-coefficient = −2.75). Forty metabolites had *P*-values below the ENT80 (effective number of independent tests accounting for 80% variance) threshold (8.27 × 10^−4^, for a complete list of results see Supplemental Table S1 in Supplementary File 2; for definition of ENT80 see Section 4.4.), of which all were inversely associated with 25(OH)D concentrations. With respect to the false discovery rate (FDR) criteria, 117 metabolites had Benjamini–Hochberg (BH) FDR [21] < 0.05, and 32 had Benjamini–Yekutieli (BY) FDR [22] < 0.05. 

### 2.2. Results from Age 3 Analysis

Four hundred and eleven children were measured for metabolomics at age 3, of whom three (0.7%) were missing plasma 25(OH)D measurements, with another missing BMI information. Therefore, at age 3, 407 VDAART children were included in the analytical sample (Table 2). A much lower proportion of children at age 3 (11.8%) had plasma 25(OH)D concentrations > 30 ng/mL compared to the age 1 samples; the average plasma 25(OH)D concentration was also lower [20.8 (SD = 8.4) ng/mL]. Overall, African American children and those from the St. Louis and Boston sites had lower mean plasma 25(OH)D concentrations. 

From the primary linear model, 21 metabolites had significant associations with plasma 25(OH)D concentration after Bonferroni correction (*P*-value threshold = 9.78 × 10^−5^), adjusting for sex, race, ethnicity, study site, age 3, BMI, season of blood collection, and asthma or recurrent wheezing status by age 3. The most significant finding was for γ-glutamylglycine, which was inversely associated with 25(OH)D concentrations (estimated β-coefficient = −2.34, *P*-value = 1.64 × 10^−8^). Fifty-three metabolites had *P*-values below the ENT80 threshold (9.67 × 10^−4^, for a complete list of results see Supplemental Table S2 in Supplementary File 2), of which 51 metabolites had negative associations, and two had positive associations with 25(OH)D concentrations. With respect to the FDR criteria, 128 metabolites had BH FDR < 0.05, and 40 had BY FDR < 0.05. 

### 2.3. Overlap between Age 1 and Age 3 Results

We focused on metabolites passing their respective ENT80 thresholds when comparing results from the age 1 samples and those from the age 3 samples (Supplemental Figure S1, Supplementary File 1). A total of 12 metabolites were significantly associated with 25(OH)D concentration at both time points with the same direction of effect (ordered by age 1 result significance): docosapentaenoate (22:5 n-6 DPA) (age 1 *P*-value = 9.74 × 10^−6^, age 3 *P*-value = 6.47 × 10^−4^), glycine (age 1 *P*-value = 3.03 × 10^−5^, age 3 *P*-value = 8.89 × 10^−4^), 1-palmitoyl-glycerophosphoethanolamine (GPE) (age 1 *P*-value = 4.22 × 10^−5^, age 3 *P*-value = 4.29 × 10^−4^), serine (age 1 *P*-value = 7.21 × 10^−5^, age 3 *P*-value = 9.43 × 10^−4^), N-acetyltaurine (age 1 *P*-value = 7.75 × 10^−5^, age 3 *P*-value = 6.96 × 10^−4^), N-palmitoylglycine (age 1 *P*-value = 1.23 × 10^−4^, age 3 *P*-value = 9.27 × 10^−5^), sphingomyelin (d18:2/16:0, d18:1/16:1) (age 1 *P*-value = 3.06 × 10^−4^, age 3 *P*-value = 1.66 × 10^−4^), arachidonate (20:4 n-6) (age 1 *P*-value = 3.10 × 10^−4^, age 3 *P*-value = 6.20 × 10^−4^), palmitoyl-linoleoyl-glycerol (16:0/18:2) (age 1 *P*-value = 5.83 × 10^−4^, age 3 *P*-value = 5.89 × 10^−4^), linoleate (18:2 n-6) (age 1 *P*-value = 7.23 × 10^−4^, age 3 *P*-value = 4.70 × 10^−5^), hydroxyproline (age 1 *P*-value = 7.38 × 10^−4^, age 3 *P*-value = 5.06 × 10^−5^), and 1-stearoyl-GPE (age 1 *P*-value = 7.44 × 10^−4^, age 3 *P*-value = 6.57 × 10^−5^), all of which were inversely associated with 25(OH)D concentrations (Table 3). Taking n-6 DPA as an example, the results can be interpreted as follows: one standard deviation increase in log_10_ n-6 DPA was associated with an average of 1.40 ng/mL decrease in plasma 25(OH)D concentrations in VDAART children at age 3, for those with the same sex, race, ethnicity, study site, BMI, season of blood collection, and asthma or recurrent wheezing status by age 3.

Noting that three of the common significant metabolites were n-6 PUFAs in the linoleic acid metabolism pathway (linoleate, arachidonate, and docosapentaenoate; for an illustration of n-6 and n-3 PUFA metabolism see Supplemental Figure S2, Supplementary File 1) [23,24]. Two additional members of this pathway, linolenate (18:3) and dihomo-linolenate (20:3), were also inversely associated with 25(OH)D, although the assay was not able to distinguish between their n-3 and n-6 forms. Both were negatively associated with 25(OH)D concentration at age 1 and age 3. Linolenate had a nominally significant association with plasma 25(OH)D concentrations at age 1 (*P*-value = 9.48 × 10^−3^), and was significantly associated with 25(OH)D concentration at age 3 (*P*-value = 1.52 × 10^−4^, below ENT80). Dihomo-linolenate had a *P*-value below ENT80 at age 1 (*P*-value = 4.40 × 10^−5^), and was nominally significantly associated with 25(OH)D concentrations at age 3 (*P*-value = 1.48 × 10^−3^). 

### 2.4. Sensitivity Analysis Results

Using Rosner’s outlier test [25], we identified six children at age 1 and one child at age 3 who were outliers in vitamin D distribution with concentrations much higher than others (Supplemental Figure S3 and S4, Supplementary File 1). We repeated the analyses after removing these outlying subjects, and the results were not substantially changed. Eleven metabolites were significantly associated with 25(OH)D concentrations at both time points with the same direction of effect (Table 4), including eight in Table 3. Based on their respective ENT80 thresholds, dihomo-linolenate (20:3) became significantly associated with 25(OH)D concentrations at both age 1 (*P*-value = 1.60 × 10^−5^) and age 3 (*P*-value = 8.10 × 10^−4^). Two additional metabolites were significant based on ENT80: linoleoyl ethanolamide and valylglycine, while sphingomyelin (d18:2/16:0, d18:1/16:1), 1-stearoyl-GPE, hydroxyproline, and palmitoyl-linoleoyl-glycerol (16:0/18:2) failed to retain significance. Removing asthma or recurrent wheezing status by age 3 from the linear models, or adding a maternal treatment group, did not substantially change the results (Supplemental Table S3, Supplementary File 2). We did not observe substantial differences in characteristics comparing children included in the analyses with those not included, except for the study site in the age 3 sample (Supplemental Table S4 and S5, Supplementary File 2), which was adjusted for in our regression models.

### 2.5. Pathway Analysis Results

**Figure 1:**
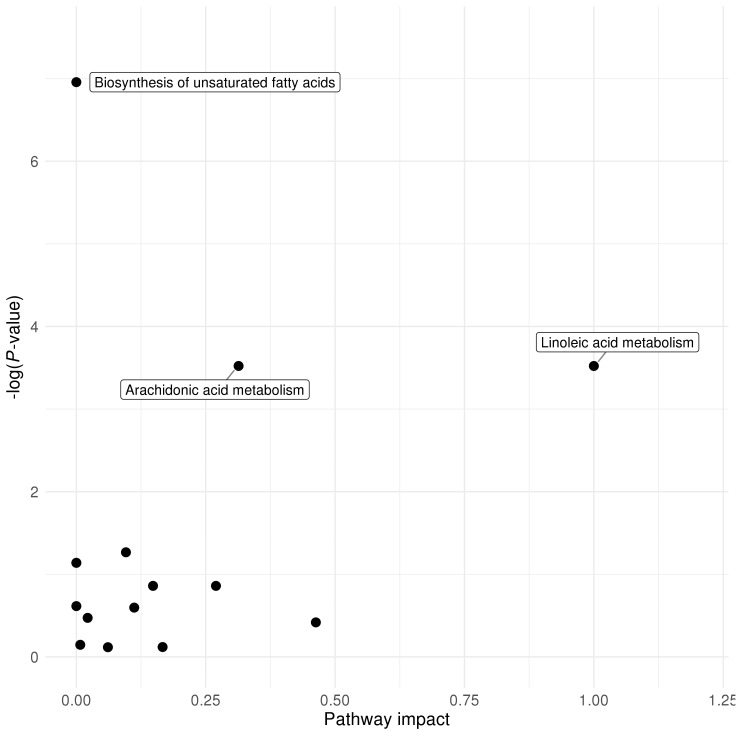
Pathway over-representation analysis significance against pathway impact plot for MetaboAnalyst pathway analysis results at age 1 (natural logarithm of *P*-value on Y-axis).

**Figure 2:**
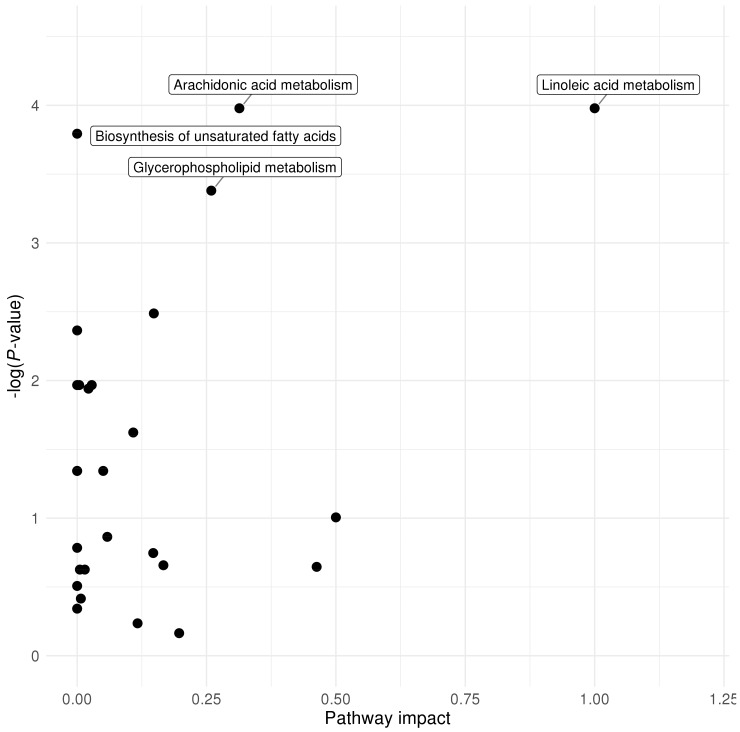
Pathway over-representation analysis significance against pathway impact plot for MetaboAnalyst pathway analysis results at age 3 (natural logarithm of *P*-value on Y-axis).

We used the web-based tool MetaboAnalyst 4.0 to perform pathway analysis of the significant metabolites (*P*-values below ENT80, for details see Materials and Methods Section 4.4.) [26]. In the age 1 samples, the arachidonic acid metabolism pathway was nominally significantly over-represented (*P*-value = 1.89 × 10^−2^), while the linoleic acid metabolism pathway had the highest pathway impact (Figure 1). Four pathways were nominally significantly over-represented among the 53 metabolites that were significant in the age 3 samples: linoleic acid metabolism (*P*-value = 1.15 × 10^−2^), arachidonic acid metabolism (*P*-value = 1.28 × 10^−2^), glycerophospholipid metabolism (*P*-value = 1.75 × 10^−2^), and methane metabolism (*P*-value = 3.57 × 10^−2^), with the highest impacts as reflected in the significance against pathway impact plot (Figure 2). Overall, the n-6 PUFA pathways, namely the linoleic acid metabolism and arachidonic acid metabolism pathways, appeared to have high impact in pathway topology analysis, and were largely over-represented in terms of association with plasma 25(OH)D concentrations.

### 2.6. Replication Analysis Results

We replicated our analysis in the Childhood Asthma Management Program (CAMP) [27] as described in Materials and Methods Section 4.5. Characteristics of CAMP children are summarized in Supplemental Table S6 and S7 in Supplementary File 2. Among the n-6 PUFAs measured in VDAART, four were relatively quantified in CAMP: linoleate, arachidonate, docosapentaenoate, and γ-linolenate (distinguished from the n-3 α form). Four other metabolites that were significant in VDAART at ages 1 and 3 were available for replication in CAMP. None of the associations with serum 25(OH)D were nominally significant at CAMP baseline. However, at the end of the trial all four n-6 PUFAs were negatively associated with 25(OH)D (same direction of estimated effects as in VDAART) with high significance: γ-linolenate *P*-value = 1.58 × 10^−4^, linoleate *P*-value = 1.65 × 10^−4^, docosapentaenoate *P*-value = 1.01 × 10^−3^, and arachidonate *P*-value = 6.10 × 10^−3^. We were also able to replicate the associations with serum 25(OH)D at the end of the CAMP trial for glycine (*P*-value = 7.43 × 10^−8^), serine (*P*-value = 3.52 × 10^−7^), hydroxyproline (*P*-value = 2.62 × 10^−5^), and linoleoyl ethanolamide (*P*-value = 7.18 × 10^−4^), with the same directions of effect as in VDAART (Supplemental Table S8 in Supplementary File 2).

## 3. Discussion

In the current study, we characterized early life plasma metabolomic profiles associated with 25(OH)D status at two time points in children of diverse racial/ethnic backgrounds. From individual-metabolite analyses, we identified twelve metabolites that were associated with 25(OH)D at both age 1 and age 3 after multiple testing correction with the ENT-based approach. Among these metabolites, three members of the n-6 long chain PUFA metabolism pathway (linoleate, arachidonate and docosapentaenoate) were replicated in CAMP, an independent cohort of older children with asthma [28]. The association between four other metabolites and 25(OH)D were also replicated. These results were further corroborated by pathway analysis where we observed high significance and/or high impact of the linoleic acid metabolism and arachidonic acid metabolism pathways. 

To our knowledge, this analysis was the first to characterize the metabolic profile of 25(OH)D concentrations at two time points in early life, with replication of the main findings in an independent population of older children. Previous studies mostly focused on adult populations [15,16,17], had metabolomics data at one single time point [15,16,17,18,19], or were in subjects with specifically defined severe illness [17,19]. In a study of 30 pregnant adolescents where half had serum 25(OH)D concentrations ≥ 20 ng/mL, Finkelstein et al. used a hierarchical mixture model to discern differences in the metabolomic profiles between low and high 25(OH)D groups, and higher leukotriene B4 levels were found to be associated with low 25(OH)D [18]. Leukotriene B4 is a downstream eicosanoid derivative of the n-6 PUFAs linoleic acid and arachidonic acid, and promotes the release of proinflammatory cytokines and reactive oxygen species (ROS) [23]. Our results for n-6 PUFAs were also consistent with findings from a recent animal study in which pregnant female rats were randomly assigned to a control or vitamin D deficient diet [29]. Compared to rats on a control diet, those on a vitamin D deficient diet had higher arachidonic acid concentrations in both plasma and liver, and higher n-6 DPA concentrations in plasma [29]. In a previous investigation in a smaller subgroup of VDAART children at age 3, Blighe et al. identified three clusters of children based on their metabolomic profiles, where the first cluster was characterized by high concentrations of fatty acids including linoleate and linolenate (18:3 n-3 or n-6), and were exposed to the lowest in utero vitamin D concentrations throughout pregnancy, while the third cluster had the opposite profile [30]. While the findings from this previous analysis identified metabolomic profiles consistent with the results presented here, they stemmed from different research questions analyzed with distinct statistical approaches, and the current analysis was based on a much larger sample size at age 3 and additional metabolomic data from another time point (age 1). 

The observed inverse associations between 25(OH)D and n-6 PUFAs in the plasma may be a reflection of the western lifestyle characterized by increased time spent indoors, sunscreen use [3], and dietary patterns with high intake of n-6 PUFAs [31]. Alternatively, a plausible mechanism has also been proposed where vitamin D can influence the metabolism of long chain PUFAs [32] (for an illustration see Supplemental Figure S5, Supplementary File 1). The n-6 PUFAs, linoleic acid and arachidonic acid are among the essential fatty acids that are important constituents of cell membrane phospholipids [33,34]. In cell culture studies, 1,25(OH)_2_D has been shown to induce gene expression of cystathionine β-synthase, which metabolizes and clears homocysteine via the transsulfuration pathway [35]. Homocysteine can function as an inducer of arachidonic acid release from the membrane and accumulation of its downstream eicosanoid and ROS [36]. In human serum samples, a recent study used metabolite set enrichment analysis to identify arachidonic acid and linoleic acid metabolism as the two pathways with significant differences comparing high- and low-homocysteine groups [37]. 1,25(OH)_2_D may also downregulate the expression of cyclooxygenase-2 (COX-2) [38], which is an enzyme that metabolizes arachidonic acid into 2-series prostaglandins and thromboxanes [23]. This suggests that high concentrations of vitamin D may help mitigate the pro-inflammatory effect of n-6 PUFAs. This anti-inflammatory pathway effect of vitamin D may in part be responsible for the reduced risk of asthma or recurrent wheezing seen in our previous pregnancy vitamin D trials [10], as arachidonic acid and its downstream eicosanoids are key in asthma airway inflammation [39]. Among metabolites of arachidonic acid, increased production of 4-series leukotrienes has been observed in subsets of patients with bronchial asthma, through increased accumulation of arachidonic acid in cells recruited in asthmatic airways (mostly eosinophils) and enhanced activity and release of enzymes in the synthesis of these eicosanoids [40]. However, n-3 PUFAs may also act as a substrate for COX-2, which derive eicosanoids that are generally considered anti-inflammatory [23]. This was also observed in our recent trial, where pregnant women were supplemented in a 2x2 factorial design with high-dose vitamin D and n-3 long chain PUFA, showing borderline interaction between the two dietary interventions with respect to asthma or recurrent wheezing in the offspring [41]. The complex interplay between vitamin D and PUFA metabolism warrants more detailed investigation.

In a cross-sectional subsample of male smokers aged 50–69 years, Nelson et al. identified three metabolites related to fish consumption (3-carboxy-4-methyl-5-propyl-2-furanpropanoate (CMPF), eicosapentaenoate (EPA), and docosahexaenoate (DHA)) that were positively associated with serum 25(OH)D concentrations, while n-6 PUFAs did not reach nominal significance [16]. We observed inverse associations between plasma EPA or DHA and 25(OH)D concentrations at both age 1 (*P*-values = 3.65 × 10^−4^ and 1.48 × 10^−3^ respectively) and age 3 (*P*-values = 3.80 × 10^−1^ and 7.84 × 10^−2^ respectively), while the directions of effect for CMPF were not consistent at the two time points (age 1 negative association *P*-value = 8.76 × 10^−3^, age 3 positive association *P*-value = 2.27 × 10^−3^). In the analysis by Nelson et al., N-acetyltaurine and 1-palmitoyl-GPE reached nominal significance and the directions of observed associations were consistent with ours. Vogt et al. discovered 30 metabolites associated with serum 25(OH)D concentrations [15], where summary measures of fatty acid unsaturation and CMPF were positively associated with 25(OH)D. The discrepancy between our results and theirs may be due to the vastly different study populations included: children in early life and middle-aged to older adults have substantial differences in lifestyle factors that may influence metabolism. As such, our results may only be generalizable to children.

Several limitations in our study should be noted. First, not all VDAART children had metabolomics measured at ages 1 and 3 years. We checked for characteristics that might be different between children included in the respective analytical samples with those not included, and only study site at age 3 was significantly different, which was adjusted for in our statistical modeling. Moreover, neither 25(OH)D concentrations nor metabolite concentrations influenced whether a child was selected for metabolomic profiling, mitigating the potential of selection bias. Second, our analyses were cross-sectional, making it difficult to establish temporality and causality between 25(OH)D concentrations and the metabolites investigated. Third, although we had sufficient sample sizes at both ages, we were likely underpowered to perform subgroup analyses. We also recognize the differences in CAMP from VDAART as a replication cohort. Different analytical platforms were used to obtain metabolomics data in serum instead of plasma, and children in CAMP were older, recruited much earlier (in the 1990s), had higher 25(OH)D concentrations overall, and all had mild-to-moderate asthma. Despite these differences, we were still able to examine common metabolites measured by both platforms based on their biochemical identities, and replicate our main findings. Finally, although we accounted for several covariates that may confound or modify the relationship between plasma metabolites and 25(OH)D concentrations, we were not able to take into consideration unmeasured factors such as diet and physical activity in early life of VDAART children. These are prone to measurement errors during infancy and toddlerhood due to individual variability and subjectivity of caregivers [42]. To address unmeasured confounding as a broader issue in metabolomics epidemiology, statistical methods have been proposed involving, e.g., latent confounding factors [43], while no agreed-upon standard approach has been established.

## 4. Materials and Methods 

### 4.1. Study Subjects

The Vitamin D Antenatal Asthma Reduction Trial (VDAART) was a randomized, double-blind, placebo-controlled trial with parallel design conducted in three centers across the United States (ClinicalTrials.gov identifier: NCT00920621). The primary aim of the trial was to determine the effect of prenatal vitamin D supplementation on the incidence of asthma outcomes in the offspring. Detailed rationale, design, methods, and results of VDAART have been published elsewhere [44,45]. Briefly, the study recruited pregnant non-smoking women aged 18–39 years between October 2009 and July 2011 [44]. At 10–18 weeks gestation, 440 women were randomized to receive 4000 IU vitamin D plus a prenatal multivitamin containing 400 IU vitamin D daily, and another 436 women were randomized to receive a placebo plus a prenatal multivitamin containing 400 IU vitamin D daily. The primary endpoint was the composite outcome of asthma or recurrent wheezing by age 3 [45]. The institutional review boards at each participating institution and the Brigham and Women’s Hospital approved protocols of the trial. All women provided written informed consent. The current analysis included children with plasma metabolomic data and 25(OH)D measurements at age 1 and age 3, respectively. Those without body mass index (BMI) measurements were further excluded since vitamin D is fat-soluble and adiposity may influence concentrations of certain metabolites, leading to final sample sizes of 450 at age 1, and 407 at age 3.

### 4.2. Vitamin D Measurements

Blood specimens were obtained from VDAART children at ages 1 and 3 years and stored following standard protocol [44]. Circulating 25(OH)D concentrations in the children’s plasma samples were measured using the DiaSorin Liaison (DiaSorin) chemiluminescence immunoassay [46]. The inter- and intra-assay coefficients of variations were 11.2% and 8.1% respectively [45]. Here we report 25(OH)D measurements in nanograms per milliliter (ng/mL). To convert 25(OH)D concentrations from nanograms per milliliter (ng/mL) to nanomoles per liter (nmol/L), multiply by 2.496.

### 4.3. Metabolomics Data

Plasma metabolites of VDAART children at ages 1 and 3 years were measured by Metabolon Inc. using both an untargeted approach and a targeted complex lipid panel. Untargeted metabolomics were profiled using ultrahigh-performance liquid chromatography coupled with tandem mass spectrometry [47,48]. The lipid panel was performed using flow injection and mass spectrometry analysis. To merge data from these two platforms, we scaled one data set to the other by a scaling factor, so that the medians of the quality control group within each data set were equivalent. We then imputed missing metabolite measures by replacement with half the lowest observed value in all samples for each metabolite. Metabolite intensities were log-10-transformed to improve their skewness. Relative quantification of 653 named and 181 unknown metabolites were processed, and 511 named metabolites with ≤ 10% missing across samples were included in this analysis. Details of the assays, data processing and quality control can be found in Supplementary File 1.

### 4.4. Statistical Analysis

Characteristics of VDAART children with metabolomic data at ages 1 and 3 are summarized in Table 1, in both the total samples, and stratified by plasma 25(OH)D concentrations (> 30 ng/mL considered desirable/sufficient and ≤ 30 ng/mL considered insufficient) [3]. Linear regression models were used to assess the associations between each plasma metabolite (as independent variable) and plasma 25(OH)D concentrations (as dependent variable), cross-sectionally at ages 1 and 3 years respectively, adjusting for potential confounding factors. Plasma metabolite intensities were standardized by autoscaling [49] so that estimated β-coefficients were on comparable scales. We visually examined the distributions of 25(OH)D concentrations at ages 1 and 3 and calculated their skewness. Rosner’s generalized extreme Studentized deviate many-outlier procedure was used to identify outliers in their distributions [25]. Sensitivity analyses excluding all outliers were then conducted to assess the robustness of our findings.

The selection of potential confounding factors was based on scientific knowledge and the literature a priori to statistical analysis, considering their causal relations with the metabolites and with plasma 25(OH)D concentrations. The primary models included sex (female, male), race (white, African American, others), ethnicity (Hispanic or Latino, not Hispanic or Latino), study site (Boston, St. Louis, San Diego), BMI (at ages 1 and 3 years respectively, continuous), and season of blood collection (four seasons separated by solstice and equinox). Having asthma or recurrent wheezing by age 3 (yes, no) was also included in the model as a potential effect modifier. These variables had no missing values within the analytical samples. We performed various sensitivity analyses to further evaluate the robustness of our findings, including the following modifications to the multivariable linear models: (1) removal of asthma or recurrent wheezing by age 3 as a covariate; (2) additional adjustment for maternal treatment group assignment (4400 IU/day or 400 IU/day vitamin D supplementation). We additionally examined the differences in these characteristics comparing children included in the respective analytical samples at ages 1 and 3 with those not included.

We considered several methods to address multiple testing. We first used Bonferroni correction where only metabolite-25(OH)D associations with *P*-values < 9.78 × 10^−5^ (0.05/511 metabolites) were considered significant, controlling the family-wise type I error rate at 0.05. Because the Bonferroni correction is overly conservative in metabolomics studies where many metabolites are highly correlated, we also used the approach based on the effective number of independent tests (ENT) [50,51]. We computed ENT as the number of principal components needed to account for 80% of the total variance in metabolites, and termed the corresponding *P*-value threshold as ENT80 (calculated using Sidak correction $1-{\left(1-0.05\right)}^{1/ENT}$). Metabolite-25(OH)D associations with *P*-values below ENT80 were declared significant. As a comparison, we also computed the false discovery rate (FDR) using the Benjamini and Hochberg (BH) procedure [21], and the more conservative Benjamini and Yekutieli (BY) procedure for multiple testing under dependency [22]. We primarily report significant metabolite-25(OH)D associations common to the age 1 and age 3 analyses with the same direction of estimated effects.

We used the web-based tool MetaboAnalyst 4.0 to perform pathway analysis of the significant metabolites (*P*-values below ENT80) [26]. All metabolites with Human Metabolome Database (HMDB) IDs were entered as the background list, and over-representation analysis was performed using the hypergeometric test, which tests if a particular group of compounds is represented more than expected by chance within the background list. In pathway topology analysis, we used relative betweenness centrality as the node importance measure, which generated pathway impact values (for more details see Supplementary File 1). 

### 4.5. Replication Analysis

To replicate our findings in an independent cohort, we used data from the Childhood Asthma Management Program (CAMP), a randomized controlled trial to evaluate the effects of different treatments (inhaled corticosteroid, inhaled non-corticosteroid, or placebo) on lung growth in children aged 5 to 12 years with mild-to-moderate asthma (ClinicalTrials.gov Identifier: NCT00000575) [27,28]. Recruitment of CAMP participants occurred between December 1993 and September 1995, and 1041 children were randomized to participate in the trial for an average of 4.3 years [28]. Approval was obtained from the institutional review boards at each of the participating institutions of CAMP. Informed consent was obtained from the children’s parents or guardians. Our replication analysis included children in CAMP with both serum 25(OH)D [52] and metabolomics data at baseline (n = 542) or end of the trial (n = 561), respectively. Metabolomic profiling in serum samples was conducted at The Broad Institute [53,54] (details in Supplementary File 1). Equivalent models to the primary analyses in VDAART were employed for 501 named metabolites, with two adjustments: (1) we removed asthma status from the models since all children in CAMP were asthmatics, and (2) we adjusted for age at sampling since CAMP was not a birth cohort. The Bonferroni threshold of 0.05/number-of-metabolites-to-replicate was used for multiple testing correction. All analyses other than the pathway analysis were conducted in R version 3.6.0 [47]. We are committed to submitting our analytical dataset to a suitable publicly accessible repository, as such time as a repository designed for epidemiological metabolomic data becomes well-established.

## 5. Conclusions

In conclusion, the current study provides evidence for an association between higher plasma 25(OH)D concentrations with lower concentrations of multiple members in the metabolic cascade of linoleic acid, an essential n-6 PUFA, in children at ages 1 and 3 years. Since both vitamin D and n-6 PUFAs are involved in inflammatory processes, these relationships warrant further investigation in other study populations of similar age.

## Figures and Tables

**Table 1:** Characteristics of VDAART children included in age 1 analysis.

	All Subjects (n = 450)	25(OH)D ≤ 30 ng/mL (n = 254)	25(OH)D > 30 ng/mL (n = 196)	*P*-Value ^1^
Age 1 25(OH)D ng/mL, mean (SD)	29.6 (10.3)	23.2 (5.2)	38.0 (9.3)	<0.001
Age 1 BMI kg/m^2^, mean (SD)	17.4 (2.2)	17.4 (1.9)	17.4 (2.5)	0.796
Sex, n (%)				0.594
Female	205 (45.6)	119 (46.9)	86 (43.9)	
Male	245 (54.4)	135 (53.1)	110 (56.1)	
Race, n (%)				0.043
African American	221 (49.1)	126 (49.6)	95 (48.5)	
Other	86 (19.1)	39 (15.4)	47 (24.0)	
White	143 (31.8)	89 (35.0)	54 (27.6)	
Ethnicity, n (%)				0.019
Hispanic or Latino	160 (35.6)	78 (30.7)	82 (41.8)	
Not Hispanic or Latino	290 (64.4)	176 (69.3)	114 (58.2)	
Study site, n (%)				0.003
Boston	140 (31.1)	71 (28.0)	69 (35.2)	
San Diego	149 (33.1)	75 (29.5)	74 (37.8)	
St. Louis	161 (35.8)	108 (42.5)	53 (27.0)	
Season of blood collection, n (%)				0.062
Spring	124 (27.6)	59 (23.2)	65 (33.2)	
Summer	108 (24.0)	63 (24.8)	45 (23.0)	
Fall	106 (23.6)	69 (27.2)	37 (18.9)	
Winter	112 (24.9)	63 (24.8)	49 (25.0)	
Asthma/wheeze by age 3, n (%)				0.669
No	309 (68.7)	177 (69.7)	132 (67.3)	
Yes	141 (31.3)	77 (30.3)	64 (32.7)	
Treatment (in pregnancy), n (%)				0.885
4400 IU/day vitamin D	229 (50.9)	126 (49.6)	95 (48.5)	
400 IU/day vitamin D	221 (49.1)	128 (50.4)	101 (51.5)	

^1^ Significance of difference was evaluated using chi-squared test for categorical variables and two-sample t-test for continuous variables. Abbreviations: BMI, body mass index; SD, standard deviation; VDAART, Vitamin D Antenatal Asthma Reduction Trial; 25(OH)D, 25-hydroxyvitamin D.

**Table 2:** Characteristics of VDAART children included in age 3 analysis.

	All Subjects (n = 407)	25(OH)D ≤ 30 ng/mL (n = 359)	25(OH)D > 30 ng/mL (n = 48)	*P*-Value ^1^
Age 3 25(OH)D ng/mL, mean (SD)	20.8 (8.4)	18.7 (6.3)	36.3 (6.1)	<0.001
Age 3 BMI kg/m^2^, mean (SD)	16.7 (1.9)	16.7 (1.9)	16.5 (1.5)	0.390
Sex, n (%)				0.243
Female	189 (46.4)	171 (47.6)	18 (37.5)	
Male	218 (53.6)	188 (52.4)	30 (62.5)	
Race, n (%)				0.002
African American	197 (48.4)	185 (51.5)	12 (25.0)	
Other	77 (18.9)	62 (17.3)	15 (31.2)	
White	133 (32.7)	112 (31.2)	21 (43.8)	
Ethnicity, n (%)				0.097
Hispanic or Latino	131 (32.2)	110 (30.6)	21 (43.8)	
Not Hispanic or Latino	276 (67.8)	249 (69.4)	27 (56.2)	
Study site, n (%)				0.020
Boston	86 (21.1)	80 (22.3)	6 (12.5)	
San Diego	140 (34.4)	115 (32.0)	25 (52.1)	
St. Louis	181 (44.5)	164 (45.7)	17 (35.4)	
Season of blood collection, n (%)				0.687
Spring	86 (21.1)	75 (20.9)	11 (22.9)	
Summer	114 (28.0)	98 (27.3)	16 (33.3)	
Fall	140 (34.4)	127 (35.4)	13 (27.1)	
Winter	67 (16.5)	59 (16.4)	8 (16.7)	
Asthma/wheeze by age 3, n (%)				1.000
No	301 (74.0)	266 (74.1)	35 (72.9)	
Yes	106 (26.0)	93 (25.9)	13 (27.1)	
Treatment (in pregnancy), n (%)				1.000
4400 IU/day vitamin D	208 (51.1)	183 (51.0)	25 (52.1)	
400 IU/day vitamin D	199 (48.9)	176 (49.0)	23 (47.9)	

^1^ Significance of difference was evaluated using chi-squared test for categorical variables and two-sample t-test for continuous variables. Abbreviations: BMI, body mass index; SD, standard deviation; VDAART, Vitamin D Antenatal Asthma Reduction Trial; 25(OH)D, 25-hydroxyvitamin D.

**Table 3:** Plasma metabolites significantly associated with 25(OH)D concentrations common to age 1 and 3 samples based on ENT80 thresholds ^1.^

Metabolite	Pathway	Age 1 Result ^2^			Age 3 Result ^3^		
		$\hat{\beta }$	*P*-Value	95% CI	$\hat{\beta }$	*P*-Value	95% CI
docosapentaenoate (n-6 DPA; 22:5)	Long Chain PUFA	−2.27	9.74 × 10^−6^	(−3.27, −1.27)	−1.40	6.47 × 10^−4^	(−2.20, −0.60)
glycine	Glycine, Serine and Threonine Metabolism	−2.06	3.03 × 10^−5^	(−3.02, −1.10)	−1.33	8.89 × 10^−4^	(−2.11, −0.55)
1-palmitoyl-GPE (16:0)	Lysophospholipid	−2.00	4.22 × 10^−5^	(−2.95, −1.05)	−1.42	4.29 × 10^−4^	(−2.20, −0.63)
serine	Glycine, Serine and Threonine Metabolism	−1.96	7.21 × 10^−5^	(−2.92, −1.00)	−1.32	9.43 × 10^−4^	(−2.11, −0.54)
N-acetyltaurine	Methionine, Cysteine, SAM and Taurine Metabolism	−1.95	7.75 × 10^−5^	(−2.90, −0.99)	−1.36	6.96 × 10^−4^	(−2.15, −0.58)
N-palmitoylglycine	Fatty Acid Metabolism (Acyl Glycine)	−1.91	1.23 × 10^−4^	(−2.88, −0.94)	−1.57	9.27 × 10^−5^	(−2.36, −0.79)
sphingomyelin (d18:2/16:0, d18:1/16:1)	Sphingomyelins	−1.80	3.06 × 10^−4^	(−2.77, −0.83)	−1.52	1.66 × 10^−4^	(−2.31, −0.74)
arachidonate (20:4 n-6)	Long Chain PUFA	−1.78	3.10 × 10^−4^	(−2.74, −0.82)	−1.40	6.20 × 10^−4^	(−2.19, −0.60)
palmitoyl-linoleoyl-glycerol (16:0/18:2)	Diacylglycerol	−1.68	5.83 × 10^−4^	(−2.64, −.073)	−1.38	5.89 × 10^−4^	(−2.16, −0.60)
linoleate (18:2 n-6)	Long Chain PUFA	−1.65	7.23 × 10^−4^	(−2.60, −0.70)	−1.65	4.70 × 10^−5^	(−2.44, −0.86)
hydroxyproline	Urea cycle; Arginine and Proline Metabolism	−1.75	7.38 × 10^−4^	(−2.77, −0.74)	−1.67	5.06 × 10^−5^	(−2.47, −0.87)
1-stearoyl-GPE (18:0)	Lysophospholipid	−1.65	7.44 × 10^−4^	(−2.61, −0.70)	−1.57	6.57 × 10^−5^	(−2.34, −0.81)

^1^ The *P*-value thresholds for declaring significance were 8.27 × 10^−4^ in age 1 analysis and 9.67 × 10^−4^ in age 3 analysis. ^2,3^ Linear models adjusted for sex, race, ethnicity, study site, BMI at respective age, season of blood collection, and asthma or recurrent wheezing status by age 3. Abbreviations: $\hat{\beta }$, estimated effect; CI, confidence interval; ENT, effective number of independent tests; GPE, glycerophosphoethanolamine; PUFA, polyunsaturated fatty acid; SAM, S-adenosyl methionine; 25(OH)D, 25-hydroxyvitamin D.

**Table 4:** Plasma metabolites significantly associated with 25(OH)D concentrations common to age 1 and 3 samples based on ENT80 thresholds ^1^ after excluding outliers in 25(OH)D distribution at respective time points.

Metabolite	Pathway	Age 1 Result ^2^			Age 3 Result ^3^		
		$\hat{\beta }$	*P*-Value	95% CI	$\hat{\beta }$	*P*-Value	95% CI
N-acetyltaurine	Methionine, Cysteine, SAM and Taurine Metabolism	−1.90	7.01 × 10^−6^	(−2.72, −1.08)	−1.37	4.05 × 10^−4^	(−2.13, −0.62)
docosapentaenoate (n-6 DPA; 22:5)	Long Chain PUFA	−1.91	1.29 × 10^−5^	(−2.77, −1.06)	−1.40	4.28 × 10^−4^	(−2.17, −0.62)
dihomo-linolenate (20:3 n-3 or n-6)	Long Chain PUFA	−1.88	1.60 × 10^−5^	(−2.72, −1.03)	−1.33	8.10 × 10^−4^	(−2.11, −0.56)
glycine	Glycine, Serine and Threonine Metabolism	−1.64	1.05 × 10^−4^	(−2.46, −0.82)	−1.38	3.67 × 10^−4^	(−2.13, −0.62)
arachidonate (20:4 n-6)	Long Chain PUFA	−1.62	1.13 × 10^−4^	(−2.44, −0.80)	−1.34	6.58 × 10^−4^	(−2.12, −0.57)
linoleate (18:2 n-6)	Long Chain PUFA	−1.56	1.79 × 10^−4^	(−2.37, −0.75)	−1.63	3.14 × 10^−5^	(−2.39, −0.87)
serine	Glycine, Serine and Threonine Metabolism	−1.59	1.87 × 10^−4^	(−2.41, −0.76)	−1.30	7.74 × 10^−4^	(−2.05, −0.55)
N-palmitoylglycine	Fatty Acid Metabolism (Acyl Glycine)	−1.50	4.28 × 10^−4^	(−2.34, −0.67)	−1.65	2.21 × 10^−5^	(−2.40, −0.89)
1-palmitoyl-GPE (16:0)	Lysophospholipid	−1.47	5.11 × 10^−4^	(−2.29, −0.64)	−1.53	8.81 × 10^−5^	(−2.28, −0.77)
linoleoyl ethanolamide	Endocannabinoid	−1.43	6.57 × 10^−4^	(−2.25, −0.61)	−1.29	9.53 × 10^−4^	(−2.06, −0.53)
valylglycine	Dipeptide	−1.44	6.59 × 10^−4^	(−2.26, −0.61)	−1.81	4.43 × 10^−6^	(−2.57, −1.04)

^1^ The *P*-value thresholds for declaring significance were 8.27 × 10^−4^ in age 1 analysis and 9.67 × 10^−4^ in age 3 analysis. ^2,3^ Linear models adjusted for sex, race, ethnicity, study site, BMI at respective age, season of blood collection, and asthma or recurrent wheezing status by age 3. Abbreviations: $\hat{\beta }$, estimated effect; CI, confidence interval; ENT, effective number of independent tests; GPE, glycerophosphoethanolamine; PUFA, polyunsaturated fatty acid; SAM, S-adenosyl methionine; 25(OH)D, 25-hydroxyvitamin D.

## Supplementary Materials

The following are available online at https://www.mdpi.com/2218-1989/10/4/151/s1, Supplementary File 1 (Supplemental Figure S1. Number of metabolites passing their respective ENT80 thresholds when comparing results from the age 1 samples and those from the age 3 samples; Supplemental Figure S2. Illustration of metabolism of n-6 and n-3 PUFA. Boxes with dashed outline indicate the enzymes of desaturation, elongation, and β-oxidation involved in the cascade. Metabolites with a check mark were included in our analyses (the metabolomics platform could not distinguish between α- and γ-linolenic acid, so linolenic acid n-6 or n-3 was reported as one metabolite). Adapted from Patterson et al. and Schmitz et al.; Supplemental Figure S3. Boxplot and histogram of 25(OH)D levels in VDAART children at age 1 before and after exclusion of outlier identified by Rosner’s outlier test; Supplemental Figure S4. Boxplot and histogram of 25(OH)D levels in VDAART children at age 3 before (upper panel) and after (lower panel) exclusion of outlier identified by Rosner’s outlier test; Supplemental Figure S5. Plausible biological mechanism of how the active form of vitamin D, 1,25-dihydroxyvitamin D [1,25(OH)2D], may influence n-6 PUFA metabolism based on literature (ROS, reactive oxygen species); Pathway topology analysis details; Metabolomic profiling in the Vitamin D Antenatal Asthma Reduction Trial; Metabolomic profiling in the Childhood Asthma Management Program); Supplementary File 2 (Supplemental Table S1. Metabolites with *P*-values below the ENT80 threshold (8.27 × 10^−4^) for their associations with plasma 25(OH)D levels in VDAART children at age 1; Supplemental Table S2. Metabolites with *P*-values below the ENT80 threshold (9.67 × 10^−4^) for their associations with plasma 25(OH)D levels in VDAART children at age 3; Supplemental Table S3. Comparison of results from primary and sensitivity analysis altering model adjustment; Supplemental Table S4. Characteristics of children according to whether they are in analytical sample at age 1; Supplemental Table S5. Characteristics of children according to whether they are in analytical sample at age 3; Supplemental Table S6. Characteristics of CAMP participants at baseline who were included in replication analysis; Supplemental Table S7. Characteristics of CAMP participants at end of trial who were included in replication analysis; Supplemental Table S8. Replication analysis results in CAMP).
