# Supplementary material for: Plasma 25-Hydroxyvitamin D Concentrations are Associated with Polyunsaturated Fatty Acid Metabolites in Young Children: Results from the Vitamin D Antenatal Asthma Reduction Trial
Source: Metabolites. 2020 Apr 14;10(4):151. doi: 10.3390/metabo10040151 (PMC7240965; doi:10.3390/metabo10040151)
Supplement: Supplementary file 1 [file metabolites-10-00151-s001.zip › Supplementary_File_1_revision1.pdf]

Title: Plasma 25-hydroxyvitamin D concentrations are associated with polyunsaturated fatty acid metabolites in young children: results from the Vitamin D Antenatal Asthma Reduction Trial

Author Names:
Mengna Huang, Rachel S. Kelly, Priyadarshini Kachroo, Su H. Chu, Kathleen Lee-Sarwar, Bo L. Chawes, Hans Bisgaard, Augusto A. Litonjua, Scott T. Weiss, Jessica Lasky-Su

Online Supplementary File 1

Contents

Supplemental figures 2

Pathway topology analysis details 7

Metabolomic profiling in the Vitamin D Antenatal Asthma Reduction Trial (VDAART) 8

Metabolomic profiling in the Childhood Asthma Management Program (CAMP) 12

References 14

Supplemental figures

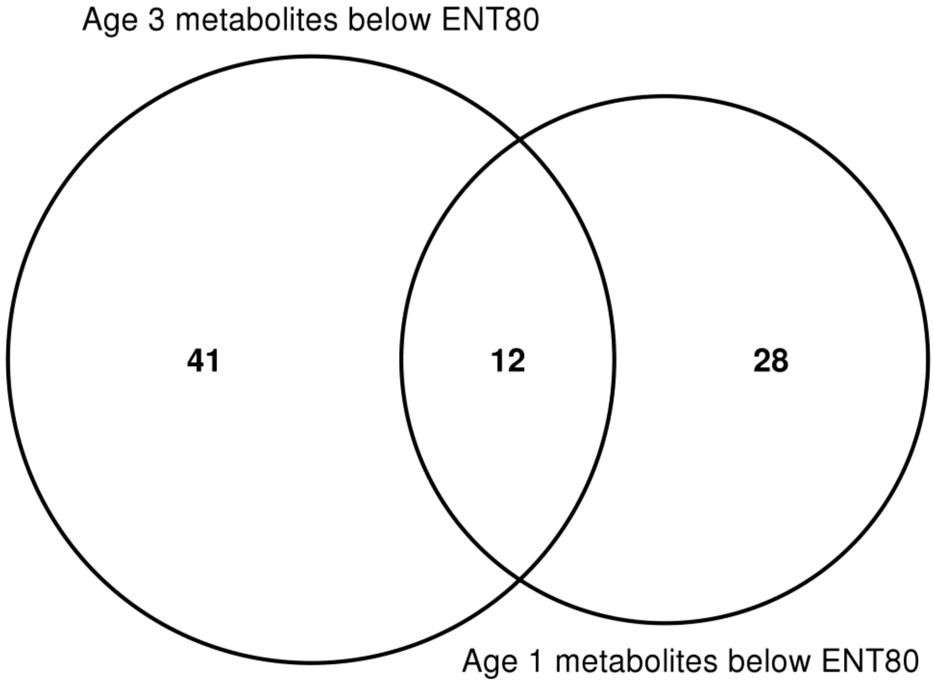

Supplemental Figure S1. Number of metabolites passing their respective ENT80 thresholds when comparing results from the age 1 samples and those from the age 3 samples.

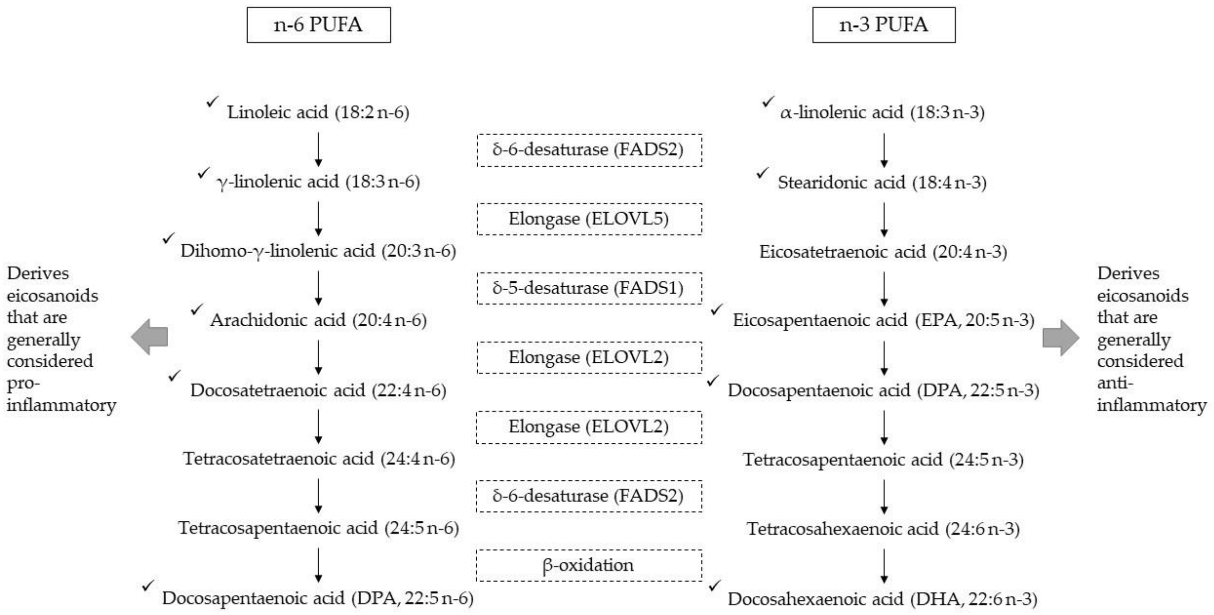

Supplemental Figure S2. Illustration of metabolism of n-6 and n-3 PUFA. Boxes with dashed outline indicate the enzymes of desaturation, elongation, and β-oxidation involved in the cascade. Metabolites with a check mark were included in our analyses (the metabolomics platform could not distinguish between α- and γ-linolenic acid, so linolenic acid n-6 or n-3 was reported as one metabolite). Adapted from Patterson et al. [1] and Schmitz et al. [2].

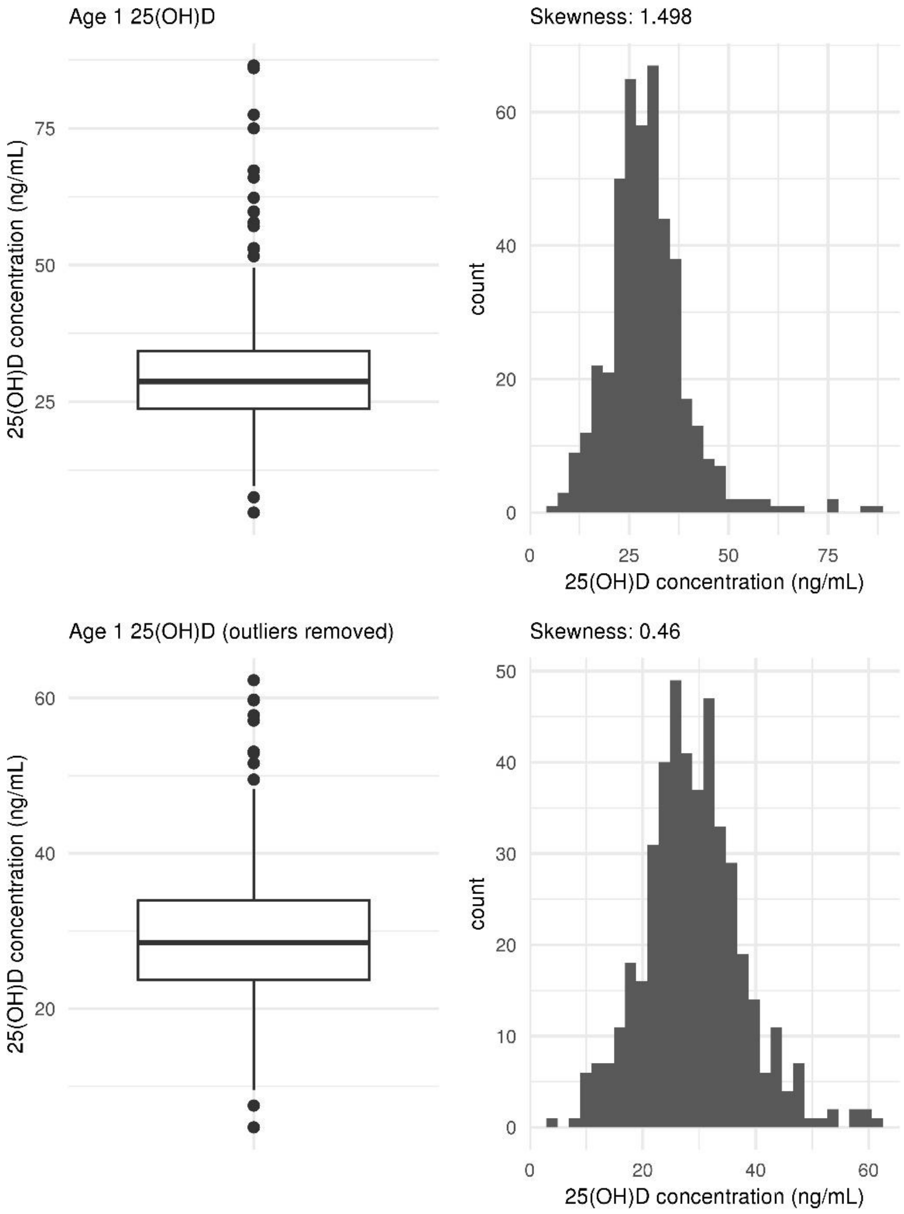

Supplemental Figure S3. Boxplot and histogram of 25(OH)D levels in VDAART children at age 1 before (upper panel) and after (lower panel) exclusion of outlier identified by Rosner's outlier test

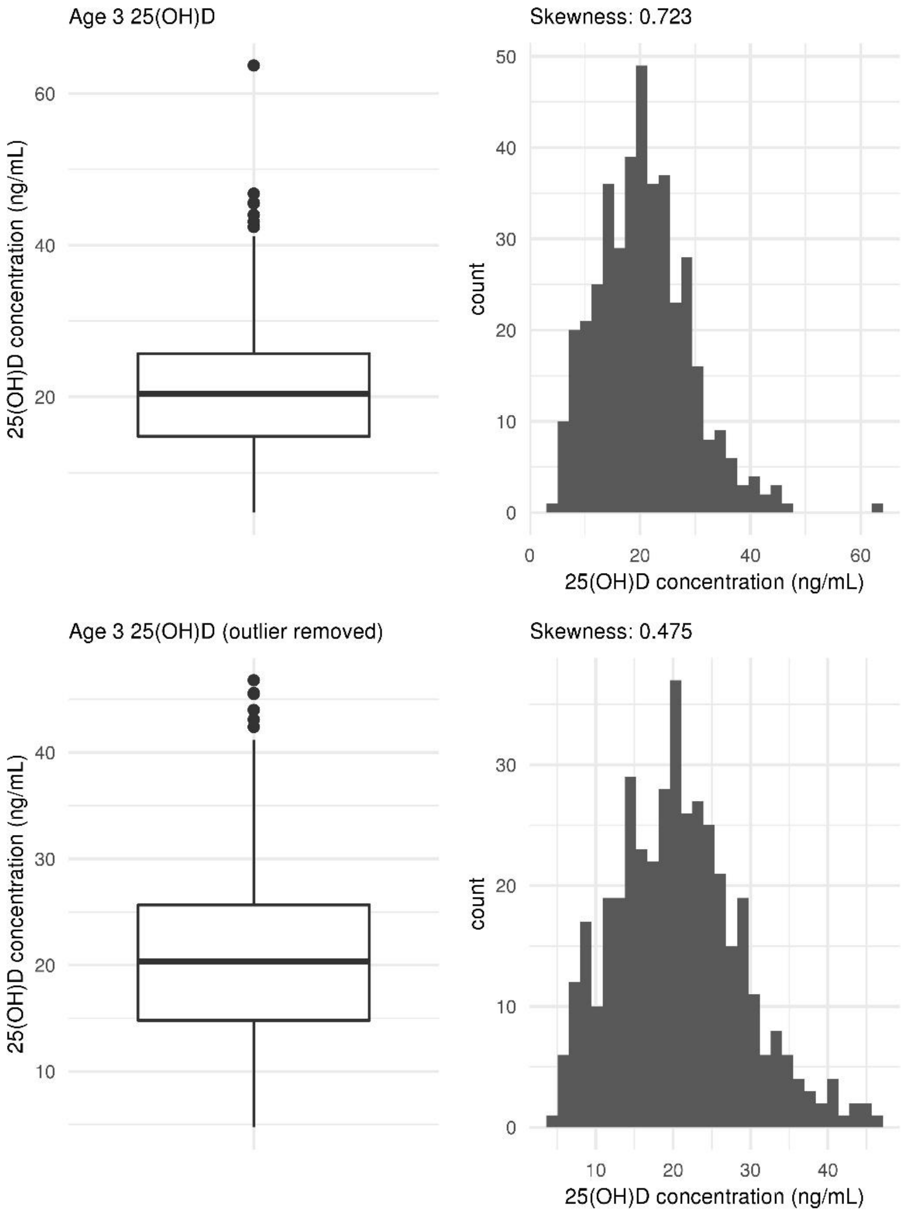

Supplemental Figure S4. Boxplot and histogram of 25(OH)D levels in VDAART children at age 3 before (upper panel) and after (lower panel) exclusion of outlier identified by Rosner's outlier test

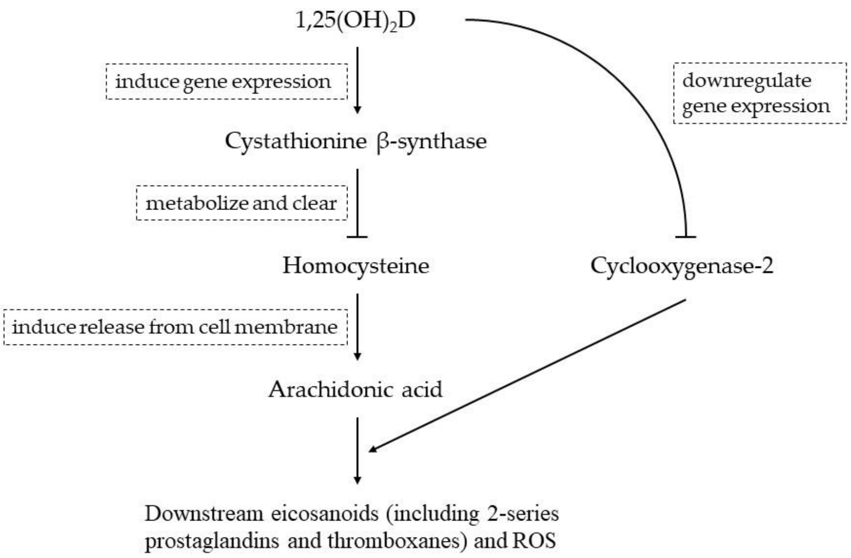

Supplemental Figure S5. Plausible biological mechanism of how the active form of vitamin D, 1,25-dihydroxyvitamin D [1,25(OH)$_2$D], may influence n-6 PUFA metabolism based on literature (ROS, reactive oxygen species).

Pathway topology analysis details

The pathway topology analysis in MetaboAnalyst 4.0 uses two well-established node centrality measures to estimate node importance - degree centrality and betweenness centrality. Degree centrality is defined as the number of links occurred upon a node. For a directed graph there are two types of degree: in-degree for links come from other nodes, and out-degree for links initiated from the current node. Metabolic networks are directed graph. Here we only consider the out-degree for node importance measure. It is assumed that nodes upstream will have regulatory roles for the downstream nodes, not vice versa. The betweenness centrality measures the number of shortest paths going through the node. Since the metabolic network is directed, we use the relative betweenness centrality for a metabolite as the importance measure. The degree centrality measure focuses more on local connectivities, while the betweenness centrality measure focuses more on global network topology.

For comparison among different pathways, the node importance values calculated from centrality measures are further normalized by the sum of the importance of the pathway. Therefore, the total/maximum importance of each pathway is 1; the importance measure of each metabolite node is actually the percentage with respect to the total pathway importance, and the pathway impact value is the cumulative percentage from the matched metabolite nodes. We selected relative betweenness centrality as the node importance measure for topological analysis.

Metabolomic profiling in the Vitamin D Antenatal Asthma Reduction Trial (VDAART)

Metabolon ultrahigh-performance liquid chromatography (UPLC) – tandem mass spectrometry (MS/MS) platform method [3]

Sample Preparation: Samples were prepared using the automated MicroLab STAR® system from Hamilton Company. Several recovery standards were added prior to the first step in the extraction process for QC purposes. To remove protein, dissociate small molecules bound to protein or trapped in the precipitated protein matrix, and to recover chemically diverse metabolites, proteins were precipitated with methanol under vigorous shaking for 2 min (Glen Mills GenoGrinder 2000) followed by centrifugation. The resulting extract was divided into five fractions: two for analysis by two separate reverse phase (RP)/UPLC-MS/MS methods with positive ion mode electrospray ionization (ESI), one for analysis by RP/UPLC-MS/MS with negative ion mode ESI, one for analysis by HILIC/UPLC-MS/MS with negative ion mode ESI, and one sample was reserved for backup. Samples were placed briefly on a TurboVap® (Zymark) to remove the organic solvent. The sample extracts were stored overnight under nitrogen before preparation for analysis.

QA/QC: Several types of controls were analyzed in concert with the experimental samples: a well-characterized, pooled matrix sample generated from a representative, large number of individuals served as a technical replicate throughout the data set; extracted water samples served as process blanks; and a cocktail of QC standards that were carefully chosen not to interfere with the measurement of endogenous compounds were spiked into every analyzed sample to allow instrument performance monitoring and aid in chromatographic alignment. Instrument variability was determined by calculating the median relative standard deviation (RSD) for the standards that were added to each sample prior to injection into the mass spectrometers. Overall process variability was determined by calculating the median RSD for all endogenous metabolites (i.e., non-instrument standards) present in 100% of the pooled matrix samples. Experimental samples were randomized across the platform run with QC samples spaced evenly among the injections.

Ultrahigh Performance Liquid Chromatography – Tandem Mass Spectrometry (UPLC-MS/MS): All methods utilized a Waters ACQUITY ultra-performance liquid chromatography (UPLC) and a Thermo Scientific Q-Exactive high resolution/accurate mass spectrometer interfaced with a heated electrospray ionization (HESI-II) source and Orbitrap mass analyzer operated at 35,000 mass resolution. The sample extract was dried then reconstituted in solvents compatible to each of the four methods. Each reconstitution solvent contained a series of standards at fixed concentrations to ensure injection and chromatographic consistency. One aliquot was analyzed using acidic positive ion conditions, chromatographically optimized for more hydrophilic compounds. In this method, the extract was gradient eluted from a C18 column (Waters UPLC BEH C18-2.1x100 mm, 1.7 µm) using water and methanol, containing 0.05% perfluoropentanoic acid (PFPA) and 0.1% formic acid (FA). Another aliquot was also analyzed using acidic positive ion conditions, however it was chromatographically optimized for more hydrophobic compounds. In this method, the extract was gradient eluted from the same afore mentioned C18 column using methanol, acetonitrile, water, 0.05% PFPA and 0.01% FA and was operated at an overall higher organic content. Another aliquot was analyzed using basic negative ion optimized conditions using a separate dedicated C18 column. The basic extracts were gradient eluted from the column using methanol and water, however with 6.5 mM Ammonium Bicarbonate at pH 8. The fourth aliquot was analyzed via negative ionization following elution from a HILIC column (Waters UPLC BEH Amide 2.1x150 mm, 1.7 µm) using a gradient consisting of water and acetonitrile with 10mM Ammonium Formate, pH 10.8. The MS analysis alternated between MS and data-dependent MSn scans using dynamic exclusion. The scan range varied slighted between methods but covered 70-1000 m/z. Raw data files were archived and extracted as described below.

Data Extraction and Compound Identification: Raw data was extracted, peak-identified and QC processed using Metabolon's hardware and software. These systems are built on a web-service platform utilizing Microsoft's .NET technologies, which run on high-performance application servers and fiber-channel storage arrays in clusters to provide active failover and load-balancing. Compounds were identified by comparison to library entries of purified standards or recurrent unknown entities. Metabolon maintains a library based on authenticated standards that contains the retention time/index (RI), mass to charge ratio (m/z), and chromatographic data (including MS/MS spectral data) on all molecules present in the library. Furthermore, biochemical identifications are based on three criteria: retention index within a narrow RI window of the proposed identification, accurate mass match to the library +/- 10 ppm, and the MS/MS forward and reverse scores between the experimental data and authentic standards. The MS/MS scores are based on a comparison of the ions present in the experimental spectrum to the ions present in the library spectrum. While there may be similarities between these molecules based on one of these factors, the use of all three data points can be utilized to distinguish and differentiate biochemicals. More than 4,500 commercially available purified standard compounds have been acquired and registered into LIMS for analysis on all platforms for determination of their analytical characteristics. Additional mass spectral entries have been created for structurally unnamed biochemicals, which have been identified by virtue of their recurrent nature (both chromatographic and mass spectral). These compounds have the potential to be identified by future acquisition of a matching purified standard or by classical structural analysis.

Curation: A variety of curation procedures were carried out to ensure that a high quality data set was made available for statistical analysis and data interpretation. The QC and curation processes were designed to ensure accurate and consistent identification of true chemical entities, and to remove those representing system artifacts, mis-assignments, and background noise. Metabolon data analysts use proprietary visualization and interpretation software to confirm the consistency of peak identification among the various samples. Library matches for each compound were checked for each sample and corrected if necessary.

Metabolite Quantification and Data Normalization: Peaks were quantified using area-under-the-curve. A data normalization step was performed to correct variation resulting from instrument inter-day tuning differences by registering the medians to equal one and normalizing each data point proportionately. For this study, two distinct sample sets were merged that contained disparity in the proportions of control group composition, precluding run day normalization. Accordingly, datasets were merged by setting the medians of the control groups in the two datasets to be equal. Because sampling was kept consistent across both sample sets, this mitigates concerns that this approach artificially decreases the variance of this control group by forcing the medians to be equal.

Metabolon complex lipid panel method

Lipids were extracted from 50 μL of plasma in the presence of deuterated internal standards using an automated BUME extraction according to the method of Lofgren et al. (J Lipid Res 2012;53(8):1690-700). Each lipid extract was divided between two glass-lined 96-well plates (70% to Plate 1 and 30% to Plate 2), then each plate was dried under nitrogen and reconstituted in 0.25mL per sample of dichloromethane:methanol (50:50) containing 10mM ammonium acetate.

Flow injection and mass spectrometry (FIA-MS) analysis was performed on a SCIEX 5500 QTRAP equipped with a SelexION Differential Mobility Separation (DMS) cell, which was operated in Multiple Reaction Monitoring (MRM) mode using both positive and negative mode electrospray in a Turbo V ion source. Plates 1 and 2 were subjected to parallel analyses, called Analysis 1 and Analysis 2, with 50 μL sample injected at a flow rate of 7 μL/min for each analysis. In Analysis 1, 472 MRM pairs corresponding to 448

endogenous lipids and 24 internal standards were monitored, comprising phosphatidylcholines, phosphatidylethanolamines, lysophosphatidylcholines, lysophosphatidylethanolamines, and phosphatidylinositols (negative ion mode), as well as sphingomyelins (positive ion mode), and the SelexION was used to apply Compensation Voltages (CoV) optimized for each lipid class, using n-propanol as the DMS modifier and the Separation Voltage set to 3500 V. In Analysis 2, 706 MRM pairs corresponding to 676 endogenous lipids and 30 internal standards were monitored, comprising free fatty acids (negative ion mode) as well as cholesteryl esters, diacylglycerols, triacylglycerols, ceramides, dihydroceramides, hexosylceramides, and lactosylceramides (positive ion mode), and the SelexION was not used. Both analyses included 20 MRM cycles with 20 msec per MRM pair, a settling time of 50 msec, and a pause between mass ranges of 5 msec. Ion source parameters are listed in the table below.

	Analysis 1		Analysis 2	
Polarity	Positive	Negative	Positive	Negative
Curtain gas	16.0	16.0	20.0	20.0
Collision gas	Medium	Medium	Medium	Medium
Ion spray voltage	4100.0	-4100.0	4100.0	-2500.0
Temperature	200.0	200.0	250.0	250.0
Ion source gas 1	17.0	17.0	20.0	20.0
Ion source gas 2	25.0	25.0	25.0	25.0

Individual lipid species were quantified based on the ratio of signal intensity for target compounds to the signal intensity for an assigned deuterated internal standard of known concentration. At least one deuterated internal standard was present per lipid class, except that phosphatidylinositols were quantified using the deuterated phosphatidylethanolamine internal standards. Lipid class concentrations were calculated from the sum of all molecular species within a class, and fatty acid compositions were determined by calculating the proportion of individual fatty acids within each class.

Data processing pipeline

Data from the UPLC-MS/MS and complex lipid panel were first merged by Metabolon using the following process: 1) make the medians of the quality control groups equivalent; 2) median scale the data to remove the run day variability with a study, then scale one of the data sets to the other data set by a scaling factor which makes the medians of these groups equivalent. In theory, the middle of these distributions should be in the same place. The large sample size which should give us good estimates of the middle of these distributions. A total of 653 named and 181 unknown metabolites were processed. We examined the metabolites by super-pathway (see table below), and calculate missingness across each metabolite and each sample. Missing values were imputed as half the minimum value across all samples for a particular metabolites. Interquartile range (IQR) and skewness were also computed (none of the metabolites had IQR = 0). Based on the distribution of skewness, it was determined that all metabolites would be log-10 transformed, and IQR and skewness were re-calculated after transformation. Principal component analysis was performed on the transformed data, and no apparent differences can be discerned by asthma status, age at sampling, or maternal treatment group.

Super-pathway	n	%
Lipid	308	36.9
Amino Acid	161	21.7
Xenobiotics	63	19.3
Peptide	36	7.6
Nucleotide	33	4.0
Cofactors and Vitamins	23	2.8
Carbohydrate	21	2.5
Energy	8	1.0
Unknown	181	21.7

Metabolomic profiling in the Childhood Asthma Management Program (CAMP)

CAMP serum metabolites were profiled using a combination of using four complimentary liquid chromatography tandem mass spectrometry (LC-MS) methods. Hydrophilic interaction liquid chromatography (HILIC) analyses of water soluble metabolites in the negative ionization mode (HILIC-neg) were conducted using an LC-MS system comprised of an AQUITY UPLC system (Waters; Milford, MA and a 5500 QTRAP mass spectrometer (SCIEX; Framingham, MA) as described previously [4]. Briefly, serum samples (30 μL) were prepared via protein precipitation with the addition of four volumes of 80% methanol containing inosine-15N4, thymine-d4 and glycocholate-d4 internal standards (Cambridge Isotope Laboratories; Andover, MA). The samples were centrifuged (10 min, 9,000 x g, 4°C), and the supernatants were injected directly onto a 150 x 2.0 mm Luna NH2 column (Phenomenex; Torrance, CA). The column was eluted at a flow rate of 400 μL/min with initial conditions of 10% mobile phase A (20 mM ammonium acetate and 20 mM ammonium hydroxide in water) and 90% mobile phase B (10 mM ammonium hydroxide in 75:25 v/v acetonitrile/methanol) followed by a 10 min linear gradient to 100% mobile phase A. MS analyses were carried out using electrospray ionization and selective multiple reaction monitoring scans in the negative ion mode. To create the method, declustering potentials and collision energies were optimized for each metabolite by infusion of reference standards. The ion spray voltage was -4.5 kV and the source temperature was 500°C.

HILIC analyses of water soluble metabolites in the positive ionization mode (HILIC-pos) were conducted using an LC-MS system comprised of a Shimadzu Nexera X2 U-HPLC (Shimadzu Corp.; Marlborough, MA) coupled to a Q Exactive hybrid quadrupole orbitrap mass spectrometer (Thermo Fisher Scientific; Waltham, MA) as described previously [5-8]. Briefly, serum samples (10 μL) were prepared via protein precipitation with the addition of nine volumes of 74.9:24.9:0.2 v/v/v acetonitrile/methanol/formic acid containing stable isotope-labeled internal standards (valine-d8, Sigma-Aldrich; St. Louis, MO; and phenylalanine-d8, Cambridge Isotope Laboratories; Andover, MA). The samples were centrifuged (10 min, 9,000 x g, 4°C), and the supernatants were injected directly onto a 150 x 2 mm, 3 μm Atlantis HILIC column (Waters; Milford, MA). The column was eluted isocratically at a flow rate of 250 μL/min with 5% mobile phase A (10 mM ammonium formate and 0.1% formic acid in water) for 0.5 minute followed by a linear gradient to 40% mobile phase B (acetonitrile with 0.1% formic acid) over 10 minutes. MS analyses were carried out using electrospray ionization in the positive ion mode using full scan analysis over 70-800 m/z at 70,000 resolution and 3 Hz data acquisition rate. Other MS settings were: sheath gas 40, sweep gas 2, spray voltage 3.5 kV, capillary temperature 350°C, S-lens RF 40, heater temperature 300°C, microscans 1, automatic gain control target 1e6, and maximum ion time 250 ms.

Positive ion mode analyses of polar and non-polar plasma lipids (C8-pos) were conducted using an LC-MS system comprised of a Shimadzu Nexera X2 U-HPLC (Shimadzu Corp.; Marlborough, MA) coupled to a Exactive Plus orbitrap mass spectrometer (Thermo Fisher Scientific; Waltham, MA) as described previsouly [5,7,8]. Serum samples (10 μL) were extracted for lipid analyses using 190 μL of isopropanol containing 1,2-didodecanoyl-sn-glycero-3-phosphocholine (Avanti Polar Lipids; Alabaster, AL). After centrifugation, supernatants were injected directly onto a 100 x 2.1 mm, 1.7 μm ACQUITY BEH C8 column (Waters; Milford, MA). The column was eluted isocratically with 80% mobile phase A (95:5:0.1 vol/vol/vol 10mM ammonium acetate/methanol/formic acid) for 1 minute followed by a linear gradient to 80% mobile-phase B (99.9:0.1 vol/vol methanol/formic acid) over 2 minutes, a linear gradient to 100% mobile phase B over 7 minutes, then 3 minutes at 100% mobile-phase B. MS analyses were carried out using electrospray ionization in the positive ion mode using full scan analysis over 200–1000 m/z at 70,000 resolution and 3 Hz data acquisition rate. Other MS settings were: sheath gas 50, in source CID 5 eV, sweep gas 5, spray voltage 3 kV, capillary temperature 300°C, S-lens RF 60, heater temperature 300°C, microscans 1, automatic

gain control target 1e6, and maximum ion time 100 ms. Lipid identities were determined based on comparison to reference plasma extracts and were denoted by total number of carbons in the lipid acyl chain(s) and total number of double bonds in the lipid acyl chain(s).

Negative ion mode analyses of free fatty acids and bile acids (C18-neg) were conducted using an LC-MS system comprised of a Shimadzu Nexera X2 U-HPLC (Shimadzu Corp.; Marlborough, MA) coupled to a Q Exactive hybrid quadrupole orbitrap mass spectrometer (Thermo Fisher Scientific; Waltham, MA). Samples were prepared using solid phase extraction. Briefly, 1.5 mL of methanol containing 1 ng/mL 15R-15-methyl PGA_2, 15S-15-methyl PGD_2, and 15S-15-methyl PGE_2 internal standards was added to 500 μL serum. The samples were centrifuged (9000 x g, 4°C, 10 minutes) and the supernatants were collected and diluted to 10 mL using water. Samples were acidified to pH 3 using 1 M hydrochloric acid and loaded onto pre-conditioned Oasis HLB solid phase extraction cartridges (12cc, 500 mg; Waters; Milford MA). The cartridges were washed with 8 mL of water and metabolites were eluted using 8 mL of methanol. Samples were dried using a TurboVap LV (Biotage) and re-suspended in 100 μL of 80% methanol containing 15R-15-methyl-PGF_{2alpha} internal standard. Extracts (10 μL) were injected onto a 150 x 2.1 mm ACQUITY BEH C18 column (Waters; Milford, MA). The column was eluted isocratically at a flow rate of 450 μL/min with 80% mobile phase A (0.01% formic acid in water) for 3 minutes followed by a linear gradient to 100% mobile phase B (0.01% acetic acid in acetonitrile) over 12 minutes. MS analyses were carried out in the negative ion mode using electrospray ionization, full scan MS acquisition over 70-850 m/z, and a resolution setting of 70,000. Metabolite identities were confirmed using authentic reference standards. Other MS settings were: sheath gas 45, sweep gas 5, spray voltage -3.5 kV, capillary temperature 320°C, S-lens RF 60, heater temperature 300°C, microscans 1, automatic gain control target 1e6, and maximum ion time 250 ms.

To evaluate data quality and enable standardization of data across the analytical queue and among batches, pooled serum reference samples were analyzed after intervals of 20 study samples. Results for each metabolite were standardized using the ratio of the value of the sample to the value of the nearest pooled reference multiplied by the median of all reference values for the metabolite. Raw data from Q Exactive/Exactive Plus instruments were processed using TraceFinder 3.3 software (Thermo Fisher Scientific; Waltham, MA) and Progenesis QI (Nonlinear Dynamics; Newcastle upon Tyne, UK) while MultiQuant 2.1 (SCIEX; Framingham, MA) was used to process 5500 QTRAP data. For each method, metabolite identities were confirmed using authentic reference standards or reference samples.

References

1. Patterson, E.; Wall, R.; Fitzgerald, G.F.; Ross, R.P.; Stanton, C. Health implications of high dietary omega-6 polyunsaturated Fatty acids. *J Nutr Metab* **2012**, *2012*, 539426, doi:10.1155/2012/539426.
2. Schmitz, G.; Ecker, J. The opposing effects of n-3 and n-6 fatty acids. *Prog Lipid Res* **2008**, *47*, 147-155, doi:10.1016/j.plipres.2007.12.004.
3. Evans, A.M.; DeHaven, C.D.; Barrett, T.; Mitchell, M.; Milgram, E. Integrated, nontargeted ultrahigh performance liquid chromatography/electrospray ionization tandem mass spectrometry platform for the identification and relative quantification of the small-molecule complement of biological systems. *Anal Chem* **2009**, *81*, 6656-6667, doi:10.1021/ac901536h.
4. Townsend, M.K.; Clish, C.B.; Kraft, P.; Wu, C.; Souza, A.L.; Deik, A.A.; Tworoger, S.S.; Wolpin, B.M. Reproducibility of metabolomic profiles among men and women in 2 large cohort studies. *Clin Chem* **2013**, *59*, 1657-1667, doi:10.1373/clinchem.2012.199133.
5. Mascanfroni, I.D.; Takenaka, M.C.; Yeste, A.; Patel, B.; Wu, Y.; Kenison, J.E.; Siddiqui, S.; Basso, A.S.; Otterbein, L.E.; Pardoll, D.M., et al. Metabolic control of type 1 regulatory T cell differentiation by AHR and HIF1-alpha. *Nat Med* **2015**, *21*, 638-646, doi:10.1038/nm.3868.
6. O'Sullivan, J.F.; Morningstar, J.E.; Yang, Q.; Zheng, B.; Gao, Y.; Jeanfavre, S.; Scott, J.; Fernandez, C.; Zheng, H.; O'Connor, S., et al. Dimethylguanidino valeric acid is a marker of liver fat and predicts diabetes. *J Clin Invest* **2017**, 10.1172/JCI95995, doi:10.1172/JCI95995.
7. Rowan, S.; Jiang, S.; Korem, T.; Szymanski, J.; Chang, M.L.; Szelog, J.; Cassalman, C.; Dasuri, K.; McGuire, C.; Nagai, R., et al. Involvement of a gut-retina axis in protection against dietary glycemia-induced age-related macular degeneration. *Proc Natl Acad Sci U S A* **2017**, *114*, E4472-E4481, doi:10.1073/pnas.1702302114.
8. Paynter, N.P.; Balasubramanian, R.; Giulianini, F.; Wang, D.D.; Tinker, L.F.; Gopal, S.; Deik, A.A.; Bullock, K.; Pierce, K.A.; Scott, J., et al. Metabolic Predictors of Incident Coronary Heart Disease in Women. *Circulation* **2018**, *137*, 841-853, doi:10.1161/CIRCULATIONAHA.117.029468.
