# Supplementary material for: Plasma 25-Hydroxyvitamin D Concentrations are Associated with Polyunsaturated Fatty Acid Metabolites in Young Children: Results from the Vitamin D Antenatal Asthma Reduction Trial
Source: Metabolites. 2020 Apr 14;10(4):151. doi: 10.3390/metabo10040151 (PMC7240965; doi:10.3390/metabo10040151)
Supplement: Supplementary file 1 [file metabolites-10-00151-s001.zip › Supplementary_File_2_revision1.pdf]

Title: Plasma 25-hydroxyvitamin D concentrations are associated with polyunsaturated fatty acid metabolites in young children: results from the Vitamin D Antenatal Asthma Reduction Trial

Author Names:
Mengna Huang, Rachel S. Kelly, Priyadarshini Kachroo, Su H. Chu, Kathleen Lee-Sarwar, Bo L. Chawes, Hans Bisgaard, Augusto A. Litonjua, Scott T. Weiss, Jessica Lasky-Su

Online Supplemental Material 2
Supplemental Tables

Contents

Supplemental Table S1. 2
Supplemental Table S2. 4
Supplemental Table S3. 6
Supplemental Table S4. 8
Supplemental Table S5. 9
Supplemental Table S6. 10
Supplemental Table S7. 11
Supplemental Table S8. 12

Supplemental Table S1. Metabolites with *P*-values below the ENT80 threshold (8.27x10^{-4}) for their associations with plasma 25(OH)D levels in VDAART children at age 1 (sorted by *P*-values)

Metabolite	Super-pathway	Sub-pathway	Estimated beta	Standard error	*P*-value	BH FDR	BY FDR
docosadienoate (22:2n6)	Lipid	Long Chain Polyunsaturated Fatty Acid (n3 and n6)	-2.75	0.48	1.93E-08	9.85E-06	6.71E-05
sphingomyelin (d18:2/24:2)	Lipid	Sphingomyelins	-2.41	0.49	1.17E-06	2.22E-04	1.52E-03
1,5-anhydroglucitol (1,5-AG)	Carbohydrate	Glycolysis, Gluconeogenesis, and Pyruvate Metabolism	-2.43	0.50	1.42E-06	2.22E-04	1.52E-03
ergothioneine	Xenobiotics	Food Component/Plant	-2.53	0.52	1.74E-06	2.22E-04	1.52E-03
docosapentaenoate (DPA; 22:5n3)	Lipid	Long Chain Polyunsaturated Fatty Acid (n3 and n6)	-2.39	0.50	2.78E-06	2.84E-04	1.93E-03
dihomolinoleate (20:2n6)	Lipid	Long Chain Polyunsaturated Fatty Acid (n3 and n6)	-2.18	0.49	9.66E-06	7.11E-04	4.84E-03
docosapentaenoate (n6 DPA; 22:5n6)	Lipid	Long Chain Polyunsaturated Fatty Acid (n3 and n6)	-2.27	0.51	9.74E-06	7.11E-04	4.84E-03
4-hydroxychlorothalonil	Xenobiotics	Chemical	-2.48	0.56	1.38E-05	8.05E-04	5.49E-03
N-acetylmethionine	Amino Acid	Methionine, Cysteine, SAM and Taurine Metabolism	-2.16	0.49	1.42E-05	8.05E-04	5.49E-03
erucate (22:1n9)	Lipid	Long Chain Monounsaturated Fatty Acid	-2.07	0.48	2.17E-05	1.11E-03	7.56E-03
glycine	Amino Acid	Glycine, Serine and Threonine Metabolism	-2.06	0.49	3.03E-05	1.41E-03	9.59E-03
1-palmitoyl-GPE (16:0)	Lipid	Lysophospholipid	-2.00	0.48	4.22E-05	1.70E-03	1.16E-02
dihomolinolenate (20:3n3 or 3n6)	Lipid	Long Chain Polyunsaturated Fatty Acid (n3 and n6)	-2.08	0.50	4.40E-05	1.70E-03	1.16E-02
1,2-dipalmitoyl-GPC (16:0/16:0)	Lipid	Phosphatidylcholine (PC)	-2.03	0.49	4.73E-05	1.70E-03	1.16E-02
dihomo-linoleoylcarnitine (C20:2)	Lipid	Fatty Acid Metabolism (Acyl Carnitine, Polyunsaturated)	-2.00	0.49	5.00E-05	1.70E-03	1.16E-02
sphingomyelin (d18:2/24:1, d18:1/24:2)	Lipid	Sphingomyelins	-2.00	0.49	5.44E-05	1.74E-03	1.18E-02
serine	Amino Acid	Glycine, Serine and Threonine Metabolism	-1.96	0.49	7.21E-05	2.17E-03	1.48E-02
N-acetyltaurine	Amino Acid	Methionine, Cysteine, SAM and Taurine Metabolism	-1.95	0.49	7.75E-05	2.20E-03	1.50E-02
2-stearoyl-GPE (18:0)	Lipid	Lysophospholipid	-1.91	0.48	9.04E-05	2.43E-03	1.66E-02
N-palmitoylglycine	Lipid	Fatty Acid Metabolism (Acyl Glycine)	-1.91	0.49	1.23E-04	3.15E-03	2.14E-02
S-methylcysteine	Amino Acid	Methionine, Cysteine, SAM and Taurine Metabolism	-1.86	0.49	1.74E-04	4.24E-03	2.89E-02
indolelactate	Amino Acid	Tryptophan Metabolism	-1.84	0.49	2.08E-04	4.69E-03	3.20E-02
palmitoylcarnitine (C16)	Lipid	Fatty Acid Metabolism (Acyl Carnitine, Long Chain Saturated)	-1.84	0.49	2.11E-04	4.69E-03	3.20E-02
2-palmitoyl-GPC* (16:0)	Lipid	Lysophospholipid	-1.81	0.49	2.31E-04	4.85E-03	3.30E-02
dihomo-linolenoylcarnitine (C20:3n3 or 6)	Lipid	Fatty Acid Metabolism (Acyl Carnitine, Polyunsaturated)	-1.83	0.49	2.37E-04	4.85E-03	3.30E-02
arachidate (20:0)	Lipid	Long Chain Saturated Fatty Acid	-1.80	0.49	2.60E-04	5.10E-03	3.48E-02

Online Supplementary File 2

Metabolite	Super-pathway	Sub-pathway	Estimated beta	Standard error	*P*-value	BH FDR	BY FDR
ornithine	Amino Acid	Urea cycle; Arginine and Proline Metabolism	-1.84	0.50	2.72E-04	5.15E-03	3.51E-02
sphingomyelin (d18:2/16:0, d18:1/16:1)	Lipid	Sphingomyelins	-1.80	0.49	3.06E-04	5.46E-03	3.72E-02
arachidonate (20:4n6)	Lipid	Long Chain Polyunsaturated Fatty Acid (n3 and n6)	-1.78	0.49	3.10E-04	5.46E-03	3.72E-02
eicosenoylcarnitine (C20:1)	Lipid	Fatty Acid Metabolism (Acyl Carnitine, Monounsaturated)	-1.76	0.49	3.43E-04	5.84E-03	3.98E-02
eicosapentaenoate (EPA; 20:5n3)	Lipid	Long Chain Polyunsaturated Fatty Acid (n3 and n6)	-1.80	0.50	3.63E-04	5.99E-03	4.08E-02
1-palmitoyl-GPC (16:0)	Lipid	Lysophospholipid	-1.72	0.49	4.47E-04	7.14E-03	4.87E-02
myristoylcarnitine (C14)	Lipid	Fatty Acid Metabolism (Acyl Carnitine, Long Chain Saturated)	-1.73	0.49	4.75E-04	7.35E-03	5.01E-02
N-palmitoyl-sphingosine (d18:1/16:0)	Lipid	Ceramides	-1.70	0.49	5.12E-04	7.69E-03	5.24E-02
palmitoyl-linoleoyl-glycerol (16:0/18:2)	Lipid	Diacylglycerol	-1.68	0.49	5.83E-04	8.52E-03	5.80E-02
5-oxoproline	Amino Acid	Glutathione Metabolism	-1.68	0.49	6.51E-04	9.24E-03	6.30E-02
stearate (18:0)	Lipid	Long Chain Saturated Fatty Acid	-1.67	0.49	7.12E-04	9.50E-03	6.47E-02
linoleate (18:2n6)	Lipid	Long Chain Polyunsaturated Fatty Acid (n3 and n6)	-1.65	0.48	7.23E-04	9.50E-03	6.47E-02
hydroxyproline	Amino Acid	Urea cycle; Arginine and Proline Metabolism	-1.75	0.52	7.38E-04	9.50E-03	6.47E-02
1-stearoyl-GPE (18:0)	Lipid	Lysophospholipid	-1.65	0.49	7.44E-04	9.50E-03	6.47E-02

Abbreviations: ENT80, effective number of independent tests that accounts for 80% of variation in metabolites; VDAART, Vitamin D Antenatal Asthma Reduction Trial; 25(OH)D, 25-hydroxyvitamin D.

Supplemental Table S2. Metabolites with *P*-values below the ENT80 threshold (9.67E-4) for their associations with plasma 25(OH)D levels in VDAART children at age 3 (sorted by *P*-values)

Metabolite	Super-pathway	Sub-pathway	Estimated beta	Standard error	*P*-value	BH FDR	BY FDR
gamma-glutamylglycine	Peptide	Gamma-glutamyl Amino Acid	-2.34	0.41	1.64E-08	8.40E-06	5.72E-05
erythronate	Carbohydrate	Aminosugar Metabolism	-1.90	0.40	2.59E-06	6.18E-04	4.21E-03
gamma-glutamylglutamate	Peptide	Gamma-glutamyl Amino Acid	-1.90	0.41	3.63E-06	6.18E-04	4.21E-03
gamma-glutamylhistidine	Peptide	Gamma-glutamyl Amino Acid	-1.85	0.40	5.39E-06	6.89E-04	4.69E-03
1-linoleoyl-GPE (18:2)	Lipid	Lysophospholipid	-1.78	0.40	1.18E-05	1.17E-03	7.95E-03
gamma-glutamylvaline	Peptide	Gamma-glutamyl Amino Acid	-1.79	0.41	1.37E-05	1.17E-03	7.95E-03
pantothenate (Vitamin B5)	Cofactors and Vitamins	Pantothenate and CoA Metabolism	1.78	0.41	2.11E-05	1.36E-03	9.27E-03
1-palmitoyl-2-linoleoyl-GPI (16:0/18:2)	Lipid	Phosphatidylinositol (PI)	-1.69	0.39	2.13E-05	1.36E-03	9.27E-03
O-sulfo-L-tyrosine	Xenobiotics	Chemical	-1.67	0.40	2.97E-05	1.69E-03	1.15E-02
1-arachidonoyl-GPE (20:4n6)	Lipid	Lysophospholipid	-1.67	0.40	3.62E-05	1.72E-03	1.17E-02
glycerophosphoethanolamine	Lipid	Phospholipid Metabolism	-1.63	0.39	3.74E-05	1.72E-03	1.17E-02
1-palmitoyl-2-linoleoyl-GPC (16:0/18:2)	Lipid	Phosphatidylcholine (PC)	-1.70	0.41	4.17E-05	1.72E-03	1.17E-02
valylglycine	Peptide	Dipeptide	-1.66	0.40	4.66E-05	1.72E-03	1.17E-02
linoleate (18:2n6)	Lipid	Long Chain Polyunsaturated Fatty Acid (n3 and n6)	-1.65	0.40	4.70E-05	1.72E-03	1.17E-02
hydroxyproline	Amino Acid	Urea cycle; Arginine and Proline Metabolism	-1.67	0.41	5.06E-05	1.72E-03	1.17E-02
1-stearoyl-GPE (18:0)	Lipid	Lysophospholipid	-1.57	0.39	6.57E-05	1.87E-03	1.28E-02
oleoyl-linoleoyl-glycerol (18:1/18:2) [1]	Lipid	Diacylglycerol	-1.60	0.40	6.76E-05	1.87E-03	1.28E-02
1-oleoyl-GPE (18:1)	Lipid	Lysophospholipid	-1.61	0.40	6.87E-05	1.87E-03	1.28E-02
gamma-tocopherol/beta-tocopherol	Cofactors and Vitamins	Tocopherol Metabolism	-1.67	0.41	6.99E-05	1.87E-03	1.28E-02
1-stearoyl-2-arachidonoyl-GPI (18:0/20:4)	Lipid	Phosphatidylinositol (PI)	-1.60	0.40	7.33E-05	1.87E-03	1.28E-02
N-palmitoylglycine	Lipid	Fatty Acid Metabolism (Acyl Glycine)	-1.57	0.40	9.27E-05	2.26E-03	1.54E-02
tyramine O-sulfate	Amino Acid	Tyrosine Metabolism	-1.60	0.41	1.04E-04	2.42E-03	1.65E-02
1-linoleoyl-GPC (18:2)	Lipid	Lysophospholipid	-1.54	0.40	1.22E-04	2.72E-03	1.85E-02
1-linoleoylglycerol (18:2)	Lipid	Monoacylglycerol	-1.56	0.41	1.36E-04	2.89E-03	1.97E-02
linolenate (18:3n3 or 3n6)	Lipid	Long Chain Polyunsaturated Fatty Acid (n3 and n6)	-1.53	0.40	1.52E-04	3.02E-03	2.06E-02
glutamate	Amino Acid	Glutamate Metabolism	-1.51	0.40	1.53E-04	3.02E-03	2.06E-02
sphingomyelin (d18:2/16:0, d18:1/16:1)	Lipid	Sphingomyelins	-1.52	0.40	1.66E-04	3.08E-03	2.10E-02
1-stearoyl-2-linoleoyl-GPC (18:0/18:2)	Lipid	Phosphatidylcholine (PC)	-1.52	0.40	1.69E-04	3.08E-03	2.10E-02

Online Supplementary File 2

Metabolite	Super-pathway	Sub-pathway	Estimated beta	Standard error	*P*-value	BH FDR	BY FDR
linoleoyl-linoleoyl-glycerol (18:2/18:2)	Lipid	Diacylglycerol	-1.48	0.39	2.07E-04	3.51E-03	2.39E-02
prolylhydroxyproline	Amino Acid	Urea cycle; Arginine and Proline Metabolism	-1.52	0.41	2.11E-04	3.51E-03	2.39E-02
1,2-dilinoleoyl-GPC (18:2/18:2)	Lipid	Phosphatidylcholine (PC)	-1.47	0.39	2.13E-04	3.51E-03	2.39E-02
1-stearoyl-2-linoleoyl-GPI (18:0/18:2)	Lipid	Phosphatidylinositol (PI)	-1.46	0.40	2.59E-04	4.08E-03	2.78E-02
1-stearoyl-2-linoleoyl-GPE (18:0/18:2)	Lipid	Phosphatidylethanolamine (PE)	-1.55	0.42	2.63E-04	4.08E-03	2.78E-02
1-linoleoyl-GPG (18:2)	Lipid	Lysophospholipid	-1.46	0.40	3.00E-04	4.51E-03	3.07E-02
cystine	Amino Acid	Methionine, Cysteine, SAM and Taurine Metabolism	1.44	0.40	3.14E-04	4.58E-03	3.12E-02
1-palmitoyl-2-linoleoyl-GPE (16:0/18:2)	Lipid	Phosphatidylethanolamine (PE)	-1.52	0.42	3.50E-04	4.96E-03	3.38E-02
N-acetylglucosamine/N-acetylgalactosamine	Carbohydrate	Aminosugar Metabolism	-1.42	0.40	4.09E-04	5.52E-03	3.76E-02
oleoyl-linoleoyl-glycerol (18:1/18:2)	Lipid	Diacylglycerol	-1.42	0.40	4.10E-04	5.52E-03	3.76E-02
1-palmitoyl-GPE (16:0)	Lipid	Lysophospholipid	-1.42	0.40	4.29E-04	5.62E-03	3.83E-02
2-aminoheptanoate	Lipid	Fatty Acid, Amino	-1.44	0.41	5.04E-04	6.44E-03	4.39E-02
palmitoyl-linoleoyl-glycerol (16:0/18:2)	Lipid	Diacylglycerol	-1.38	0.40	5.89E-04	7.35E-03	5.01E-02
arachidonate (20:4n6)	Lipid	Long Chain Polyunsaturated Fatty Acid (n3 and n6)	-1.40	0.40	6.20E-04	7.54E-03	5.14E-02
docosapentaenoate (n6 DPA; 22:5n6)	Lipid	Long Chain Polyunsaturated Fatty Acid (n3 and n6)	-1.40	0.41	6.47E-04	7.58E-03	5.17E-02
palmitate (16:0)	Lipid	Long Chain Saturated Fatty Acid	-1.39	0.40	6.56E-04	7.58E-03	5.17E-02
1-(1-enyl-palmitoyl)-GPE (P-16:0)	Lipid	Lysoplasmalogen	-1.35	0.39	6.68E-04	7.58E-03	5.17E-02
N-acetyltaurine	Amino Acid	Methionine, Cysteine, SAM and Taurine Metabolism	-1.36	0.40	6.96E-04	7.74E-03	5.27E-02
oleate/vaccenate (18:1)	Lipid	Long Chain Monounsaturated Fatty Acid	-1.35	0.40	8.30E-04	9.00E-03	6.13E-02
1-stearoyl-GPC (18:0)	Lipid	Lysophospholipid	-1.33	0.39	8.46E-04	9.00E-03	6.13E-02
cholesterol	Lipid	Sterol	-1.35	0.40	8.82E-04	9.00E-03	6.13E-02
glycine	Amino Acid	Glycine, Serine and Threonine Metabolism	-1.33	0.40	8.89E-04	9.00E-03	6.13E-02
1-(1-enyl-palmitoyl)-2-linoleoyl-GPC (P-16:0/18:2)	Lipid	Plasmalogen	-1.41	0.42	8.98E-04	9.00E-03	6.13E-02
serine	Amino Acid	Glycine, Serine and Threonine Metabolism	-1.32	0.40	9.43E-04	9.15E-03	6.24E-02
1-linoleoyl-2-linolenoyl-GPC (18:2/18:3)	Lipid	Phosphatidylcholine (PC)	-1.34	0.40	9.49E-04	9.15E-03	6.24E-02

Abbreviations: ENT80, effective number of independent tests that accounts for 80% of variation in metabolites; VDAART, Vitamin D Antenatal Asthma Reduction Trial; 25(OH)D, 25-hydroxyvitamin D.

Supplemental Table S3. Comparison of results from primary and sensitivity analysis altering model adjustment

		Age 1 results					
		**Primary model results**		**Sensitivity 1 results**		**Sensitivity 2 results**	
Metabolite	Sub-pathway	Estimated beta	*P*-value	Estimated beta	*P*-value	Estimated beta	*P*-value
docosapentaenoate (n6 DPA; 22:5n6)	Long Chain Polyunsaturated Fatty Acid (n3 and n6)	-2.27	9.74E-06	-2.29	7.12E-06	-2.28	9.11E-06
glycine	Glycine, Serine and Threonine Metabolism	-2.06	3.03E-05	-2.04	3.57E-05	-2.07	2.71E-05
1-palmitoyl-GPE (16:0)	Lysophospholipid	-2.00	4.22E-05	-2.00	4.28E-05	-2.00	4.42E-05
serine	Glycine, Serine and Threonine Metabolism	-1.96	7.21E-05	-1.95	7.56E-05	-1.95	8.26E-05
N-acetyltaurine	Methionine, Cysteine, SAM and Taurine Metabolism	-1.95	7.75E-05	-1.96	7.05E-05	-1.94	8.17E-05
N-palmitoylglycine	Fatty Acid Metabolism (Acyl Glycine)	-1.91	1.23E-04	-1.93	1.09E-04	-1.92	1.21E-04
sphingomyelin (d18:2/16:0, d18:1/16:1)	Sphingomyelins	-1.80	3.06E-04	-1.81	2.73E-04	-1.80	3.10E-04
arachidonate (20:4n6)	Long Chain Polyunsaturated Fatty Acid (n3 and n6)	-1.78	3.10E-04	-1.79	2.78E-04	-1.77	3.34E-04
palmitoyl-linoleoyl-glycerol (16:0/18:2)	Diacylglycerol	-1.68	5.83E-04	-1.69	5.51E-04	-1.68	5.95E-04
linoleate (18:2n6)	Long Chain Polyunsaturated Fatty Acid (n3 and n6)	-1.65	7.23E-04	-1.67	6.07E-04	-1.65	7.50E-04
hydroxyproline	Urea cycle; Arginine and Proline Metabolism	-1.75	7.38E-04	-1.75	7.46E-04	-1.79	5.80E-04
1-stearoyl-GPE (18:0)	Lysophospholipid	-1.65	7.44E-04	-1.65	7.33E-04	-1.64	7.99E-04

		Age 3 results					
		**Primary model results**		**Sensitivity 1 results**		**Sensitivity 2 results**	
Metabolite	Sub-pathway	Estimated beta	*P*-value	Estimated beta	*P*-value	Estimated beta	*P*-value
docosapentaenoate (n6 DPA; 22:5n6)	Long Chain Polyunsaturated Fatty Acid (n3 and n6)	-1.40	6.47E-04	-1.38	6.33E-04	-1.42	5.90E-04
glycine	Glycine, Serine and Threonine Metabolism	-1.33	8.89E-04	-1.33	8.63E-04	-1.38	6.71E-04

1-palmitoyl-GPE (16:0)	Lysophospholipid	-1.42	4.29E-04	-1.42	4.00E-04	-1.47	3.28E-04
serine	Glycine, Serine and Threonine Metabolism	-1.32	9.43E-04	-1.32	9.36E-04	-1.36	7.58E-04
N-acetyltaurine	Methionine, Cysteine, SAM and Taurine Metabolism	-1.36	6.96E-04	-1.36	7.03E-04	-1.36	7.04E-04
N-palmitoylglycine	Fatty Acid Metabolism (Acyl Glycine)	-1.57	9.27E-05	-1.58	8.75E-05	-1.58	9.15E-05
sphingomyelin (d18:2/16:0, d18:1/16:1)	Sphingomyelins	-1.52	1.66E-04	-1.53	1.55E-04	-1.53	1.63E-04
arachidonate (20:4n6)	Long Chain Polyunsaturated Fatty Acid (n3 and n6)	-1.40	6.20E-04	-1.39	5.79E-04	-1.40	5.94E-04
palmitoyl-linoleoyl-glycerol (16:0/18:2)	Diacylglycerol	-1.38	5.89E-04	-1.38	5.51E-04	-1.40	5.33E-04
linoleate (18:2n6)	Long Chain Polyunsaturated Fatty Acid (n3 and n6)	-1.65	4.70E-05	-1.65	4.37E-05	-1.65	4.62E-05
hydroxyproline	Urea cycle; Arginine and Proline Metabolism	-1.67	5.06E-05	-1.67	4.89E-05	-1.68	4.70E-05
1-stearoyl-GPE (18:0)	Lysophospholipid	-1.57	6.57E-05	-1.58	6.11E-05	-1.60	5.55E-05

Results for metabolites passing ENT80 threshold at both age 1 and age 3 in primary analysis (Table 3) are listed here
Sensitivity 1: removing asthma or recurrent wheezing status by age 3 from the primary analysis model
Sensitivity 2: adding maternal treatment group to the primary analysis model

Supplemental Table S4. Characteristics of children according to whether they are in analytical sample at age 1

		Not in analytical sample (n = 356)	In analytical sample (n = 450)	*P*-value[1]
Sex, n (%)				0.180
	Female	180 (50.6)	205 (45.6)	
	Male	176 (49.4)	245 (54.4)	
Race, n (%)				0.755
	African American	169 (47.5)	221 (49.1)	
	Other	65 (18.3)	86 (19.1)	
	White	122 (34.3)	143 (31.8)	
Ethnicity, n (%)				0.289
	Hispanic or Latino	113 (31.7)	160 (35.6)	
	Not Hispanic or Latino	243 (68.3)	290 (64.4)	
Study site, n (%)				0.637
	Boston	100 (28.1)	140 (31.1)	
	San Diego	125 (35.1)	149 (33.1)	
	St. Louis	131 (36.8)	161 (35.8)	
Treatment (maternal), n (%)				0.513
	4400 IU/day vitamin D	184 (51.7)	221 (49.1)	
	400 IU/day vitamin D	172 (48.3)	229 (50.9)	
Asthma/wheeze by age 3, n (%)[2]				0.124
	No	221 (74.2)	309 (68.7)	
	Yes	77 (25.8)	141 (31.3)	
BMI kg/m^2, mean (SD)[2]		17.7 (2.0)	17.4 (2.2)	0.065

[1] Significance of difference was evaluated using chi-squared test for categorical variables and two-sample t-test for continuous variables.

[2] In those not included in analytical sample at age 1, 58 children were missing asthma/wheeze by age 3; 102 were missing BMI.

Abbreviations: BMI, body mass index; SD, standard deviation.

Supplemental Table S5. Characteristics of children according to whether they are in analytical sample at age 3

		Not in analytical sample (n = 399)	In analytical sample (n = 407)	*P*-value[1]
Sex, n (%)				0.489
	Female	196 (49.1)	189 (46.4)	
	Male	203 (50.9)	218 (53.6)	
Race, n (%)				0.988
	African American	193 (48.4)	197 (48.4)	
	White	132 (33.1)	133 (32.7)	
	Other	74 (18.5)	77 (18.9)	
Ethnicity, n (%)				0.344
	Hispanic or Latino	142 (35.6)	131 (32.2)	
	Not Hispanic or Latino	257 (64.4)	276 (67.8)	
Study site, n (%)				<0.001
	San Diego	134 (33.6)	140 (34.4)	
	Boston	154 (38.6)	86 (21.1)	
	St. Louis	111 (27.8)	181 (44.5)	
Treatment (maternal), n (%)				0.674
	4400 IU/day vitamin D	197 (49.4)	208 (51.1)	
	400 IU/day vitamin D	202 (50.6)	199 (48.9)	
Asthma/wheeze by age 3, n (%)[2]				0.050
	No	229 (67.2)	301 (74.0)	
	Yes	112 (32.8)	106 (26.0)	
BMI kg/m2, mean (SD)[2]		16.6 (1.5)	16.7 (1.9)	0.594

[1] Significance of difference was evaluated using chi-squared test for categorical variables and two-sample t-test for continuous variables.

[2] In those not included in analytical sample at age 3, 58 children were missing asthma/wheeze by age 3; 149 were missing BMI.

Abbreviations: BMI, body mass index; SD, standard deviation.

Supplemental Table S6. Characteristics of CAMP participants at baseline who were included in replication analysis

	All subjects (n = 542)	25(OH)D ≤ 30 ng/mL (n = 195)	25(OH)D > 30 ng/mL (n = 347)	*P*-value[1]
25(OH)D ng/mL, mean (SD)	37.9 (15.6)	23.1 (5.4)	46.2 (13.0)	<0.001
Age at baseline, mean (SD)	8.8 (2.1)	9.2 (2.2)	8.6 (2.1)	0.003
Sex, n (%)				0.500
Female	197 (36.3)	75 (38.5)	122 (35.2)	
Male	345 (63.7)	120 (61.5)	225 (64.8)	
Race/ethnicity, n (%)				<0.001
Non-Hispanic white	379 (69.9)	106 (54.4)	273 (78.7)	
Non-Hispanic black	81 (14.9)	54 (27.7)	27 (7.8)	
Hispanic	56 (10.3)	20 (10.3)	36 (10.4)	
Other	26 (4.8)	15 (7.7)	11 (3.2)	
Treatment group, n (%)				0.127
Budesonide	147 (27.1)	45 (23.1)	102 (29.4)	
Nedocromil	166 (30.6)	69 (35.4)	97 (28.0)	
Placebo	229 (42.3)	81 (41.5)	148 (42.7)	
Study clinic, n (%)				<0.001
1	67 (12.4)	15 (7.7)	52 (15.0)	
2	62 (11.4)	29 (14.9)	33 (9.5)	
3	44 (8.1)	22 (11.3)	22 (6.3)	
4	68 (12.5)	19 (9.7)	49 (14.1)	
5	61 (11.3)	16 (8.2)	45 (13.0)	
6	66 (12.2)	29 (14.9)	37 (10.7)	
7	103 (19.0)	30 (15.4)	73 (21.0)	
8	71 (13.1)	35 (17.9)	36 (10.4)	
Season of blood draw, n (%)				<0.001
Spring	214 (39.5)	90 (46.2)	124 (35.7)	
Summer	128 (23.6)	25 (12.8)	103 (29.7)	
Fall	81 (14.9)	28 (14.4)	53 (15.3)	
Winter	119 (22.0)	52 (15.3)	67 (19.3)	
BMI at baseline kg/m^2, mean (SD)	18.0 (3.3)	18.6 (3.6)	17.6 (3.1)	0.001

[1] Significance of difference was evaluated using chi-squared test for categorical variables and two-sample t-test for continuous variables.

Abbreviations: BMI, body mass index; CAMP, Childhood Asthma Management Program; SD, standard deviation; 25(OH)D, 25-hydroxyvitamin D.

Supplemental Table S7. Characteristics of CAMP participants at end of trial who were included in replication analysis

	All subjects (n = 561)	25(OH)D ≤ 30 ng/ml (n = 318)	25(OH)D > 30 ng/ml (n = 243)	*P*-value[1]
25(OH)D ng/ml, mean (SD)	30.4 (14.5)	20.6 (6.1)	43.2 (12.2)	<0.001
Age at end of trial, mean (SD)	12.8 (2.2)	13.0 (2.1)	12.5 (2.2)	0.015
Sex, n (%)				0.024
Female	206 (36.7)	130 (40.9)	76 (31.3)	
Male	355 (63.3)	188 (59.1)	167 (68.7)	
Race/ethnicity, n (%)				<0.001
Non-Hispanic white	397 (70.8)	197 (61.9)	200 (82.3)	
Non-Hispanic black	81 (14.4)	68 (21.4)	13 (5.3)	
Hispanic	56 (10.0)	39 (12.3)	17 (7.0)	
Other	27 (4.8)	14 (4.4)	13 (5.3)	
Treatment group, n (%)				0.748
Budesonide	155 (27.6)	84 (26.4)	71 (29.2)	
Nedocromil	170 (30.3)	97 (30.5)	73 (30.0)	
Placebo	236 (42.1)	137 (43.1)	99 (40.7)	
Study clinic, n (%)				<0.001
1	68 (12.1)	41 (12.9)	27 (11.1)	
2	64 (11.4)	45 (14.2)	19 (7.8)	
3	44 (7.8)	35 (11.0)	9 (3.7)	
4	71 (12.7)	49 (15.4)	22 (9.1)	
5	61 (10.9)	13 (4.1)	48 (19.8)	
6	77 (13.7)	41 (12.9)	36 (14.8)	
7	104 (18.5)	50 (15.7)	54 (22.2)	
8	72 (12.8)	44 (13.8)	28 (11.5)	
Season of blood draw, n (%)				<0.001
Spring	228 (40.6)	139 (43.7)	89 (36.6)	
Summer	124 (22.1)	40 (12.6)	84 (34.6)	
Fall	90 (16.0)	60 (18.9)	30 (12.3)	
Winter	119 (21.2)	79 (24.8)	40 (16.5)	
BMI at end of trial kg/m^2, mean (SD)	21.2 (4.5)	21.8 (4.8)	20.4 (3.9)	<0.001

[1] Significance of difference was evaluated using chi-squared test for categorical variables and two-sample t-test for continuous variables.

Abbreviations: BMI, body mass index; CAMP, Childhood Asthma Management Program; SD, standard deviation; 25(OH)D, 25-hydroxyvitamin D.

Supplemental Table S8. Replication analysis results in CAMP (sorted by end-of-trial results *P*-values)

Metabolite	Baseline results			End-of-trial results		
	Estimated beta	Standard error	*P*-value	Estimated beta	Standard error	*P*-value
glycine	-0.36	0.64	5.71E-01	-2.95	0.54	7.43E-08
serine	-0.59	0.66	3.74E-01	-2.94	0.57	3.52E-07
hydroxyproline	-0.09	0.63	8.81E-01	-2.16	0.51	2.62E-05
γ-linolenate	-0.88	0.64	1.74E-01	-2.18	0.57	1.58E-04
linoleate	-0.50	0.66	4.52E-01	-2.14	0.56	1.65E-04
linoleoyl ethanolamide	0.71	0.64	2.62E-01	-1.91	0.56	7.18E-04
docosapentaenoate	-0.52	0.64	4.19E-01	-1.88	0.57	1.01E-03
arachidonate	-0.45	0.72	5.30E-01	-1.64	0.59	6.10E-03

Abbreviations: CAMP, Childhood Asthma Management Program.
